# Macrophages in Colorectal Cancer: from Normal Mucosa to Distant Metastasis: Beyond the M1/M2 Paradigm

**DOI:** 10.7150/jca.126772

**Published:** 2026-01-01

**Authors:** Sergii Pavlov, Esraa Ali, Filip Ambrozkiewicz, Wenjing Ye, Marie Rajtmajerová, Václav Liška, Kari Hemminki, Andriy Trailin

**Affiliations:** 1 Laboratory of Translational Cancer Genomics, Biomedical Center, Faculty of Medicine in Pilsen, Charles University, Alej Svobody 1665/76, 32300 Pilsen, Czech Republic.; 2 Laboratory of Cancer Treatment and Tissue Regeneration, Biomedical Center, Faculty of Medicine in Pilsen, Charles University, Alej Svobody 1665/76, 32300 Pilsen, Czech Republic.; 3 Department of Surgery and Biomedical Center, Faculty of Medicine in Pilsen, Charles University, Alej Svobody 80, 32300 Pilsen, Czech Republic.; 4 Department of Cancer Epidemiology, German Cancer Research Center, Im Neuenheimer Feld 280, 69120 Heidelberg, Germany.

**Keywords:** colorectal cancer, tumor-associated macrophages, M1/M2 markers, tumor microenvironment, normal mucosa, adenoma-colorectal cancer-liver metastasis sequence, prognostic significance.

## Abstract

Colorectal cancer (CRC) is the third most common malignancy and leading cause of mortality worldwide. Tumor microenvironment (TME) strongly influences CRC growth, immune evasion, and metastasis. Among various immune cells, tumor-associated macrophages (TAMs) act as key regulators of cancer progression. Although traditionally classified as M1 (pro-inflammatory, anti-tumor) or M2 (anti-inflammatory, pro-tumor), single-cell RNA sequencing and spatial transcriptomics have revealed that macrophage phenotypes exist along a continuum, challenging the classic dichotomy.

This review investigates macrophages throughout CRC development, from normal mucosa to adenoma, primary tumor, and liver metastasis. Early adenomas feature M1-like macrophages that drive local inflammation, whereas advanced adenomas and invasive CRC comprise M2-like macrophages promoting angiogenesis, extracellular matrix remodeling, and immunosuppression.

TAMs are crucial in CRC metastasis, particularly to the liver. M2-polarized Kupffer cells express CD206 and CD163, secrete hepatocyte growth factor, and activate PI3K/AKT signaling, thus aiding extravasation, survival, and proliferation of metastatic cells. They also foster lymphangiogenesis and immunosuppression through release of IL-10 and TGF-β.

CRC's consensus molecular subtype (CMS) impacts the profile of TAMs: CMS1 (microsatellite instability-high) tumors typically harbor an anti-tumor M1 macrophages, while CMS4 (mesenchymal) tumors are enriched in M2-like TAMs, which facilitate stromal remodeling and angiogenesis, ultimately contributing to a poor prognosis.

Spatial distribution also matters. Abundant M1 macrophages at the invasive margin correlate with better outcomes, whereas M2 macrophages in tumor centers and metastatic sites drive disease progression. Some CD206+ macrophages, however, support vascular normalization, which can limit metastasis. These findings underscore the complexity of TAMs in CRC and highlight the necessity of multi-marker phenotyping.

Given the limitations of the M1/M2 paradigm, advanced techniques such as spatial transcriptomics and single-cell RNA sequencing offer novel insights into TAM heterogeneity. Future therapeutic strategies targeting TAMs, including metabolic reprogramming, epigenetic modulators, and immune checkpoint inhibitors, hold promise for improving CRC patient outcomes by shifting the balance toward an anti-tumor immune response.

## 1. Introduction

CRC is the third most common type of cancer and the second leading cause of cancer-related mortality in the world 1. Distant CRC metastases, which most frequently appear in the liver, drastically worsen survival 2. Emerging evidence suggests that both adaptive and innate immune cells in the TME play a critical role in CRC development and progression 3.

Macrophages are a common component of CRC TME. As antigen-presenting cells (APCs), macrophages present tumor antigens on their surface through the major histocompatibility complex class II (MHC II), promoting the activation and differentiation of T cells, interconnecting innate and adaptive immunity. As effectors, they destroy cancer cells by enzymatic degradation via lysosomal enzymes and the generation of reactive oxygen species 4. In addition, they produce cytokines and chemokines to slow down tumor progression either directly or by modulating other immune cells 4.

The concept of TAMs was introduced to describe the role of macrophages 5 in tumor-induced immunosuppression, invasion and metastasis. TAMs include M0 non-polarized macrophages, which, in response to signals from ТME, acquire M1, M2 and other more complex phenotypes 4. Unlike other macrophages, TAMs have a higher proliferative capacity and are characterized by both M2 (predominantly) and M1-like transcriptional profiles. TAMs can exhibit both pro-tumor (M2-like) and anti-tumor (M1-like) activities depending on their polarization states and the signals they receive from the TME 6, 7, 8, 9. This diversity is reflected in the expression of various surface markers and functional characteristics, which ultimately shape their biological roles 4, 5.

Considering the phenotypic diversity of TAMs and the presence of transitional forms between individual types, classification based on the M1/M2 dichotomy is somewhat outdated, making it challenging to describe the diverse biological functions performed by TAMs. This may account for the conflicting data on the anti-tumor and pro-tumor roles of M1 and M2 macrophages. Accordingly, it is important to investigate the biological functions of specific macrophage molecules, their expression levels, and potential co-expression with other markers to better characterize the effects of TAMs 10, 11.

The widespread use of single-cell RNA sequencing in recent years has contributed to accumulation of data illustrating cellular heterogeneity of tumor tissue. New associations have been established between the molecular diversity of TAMs and their functional roles 12, 13.

The current review focuses on the phenotypically driven diversity of TAMs biological effects in CRC development, progression, and metastasis. Additionally, it aims to analyze data regarding the specificity of individual markers for different macrophage phenotypes and their biological roles in various regions of primary tumors and metastases.

## 2. Diversity of macrophages

**Figure 1:**
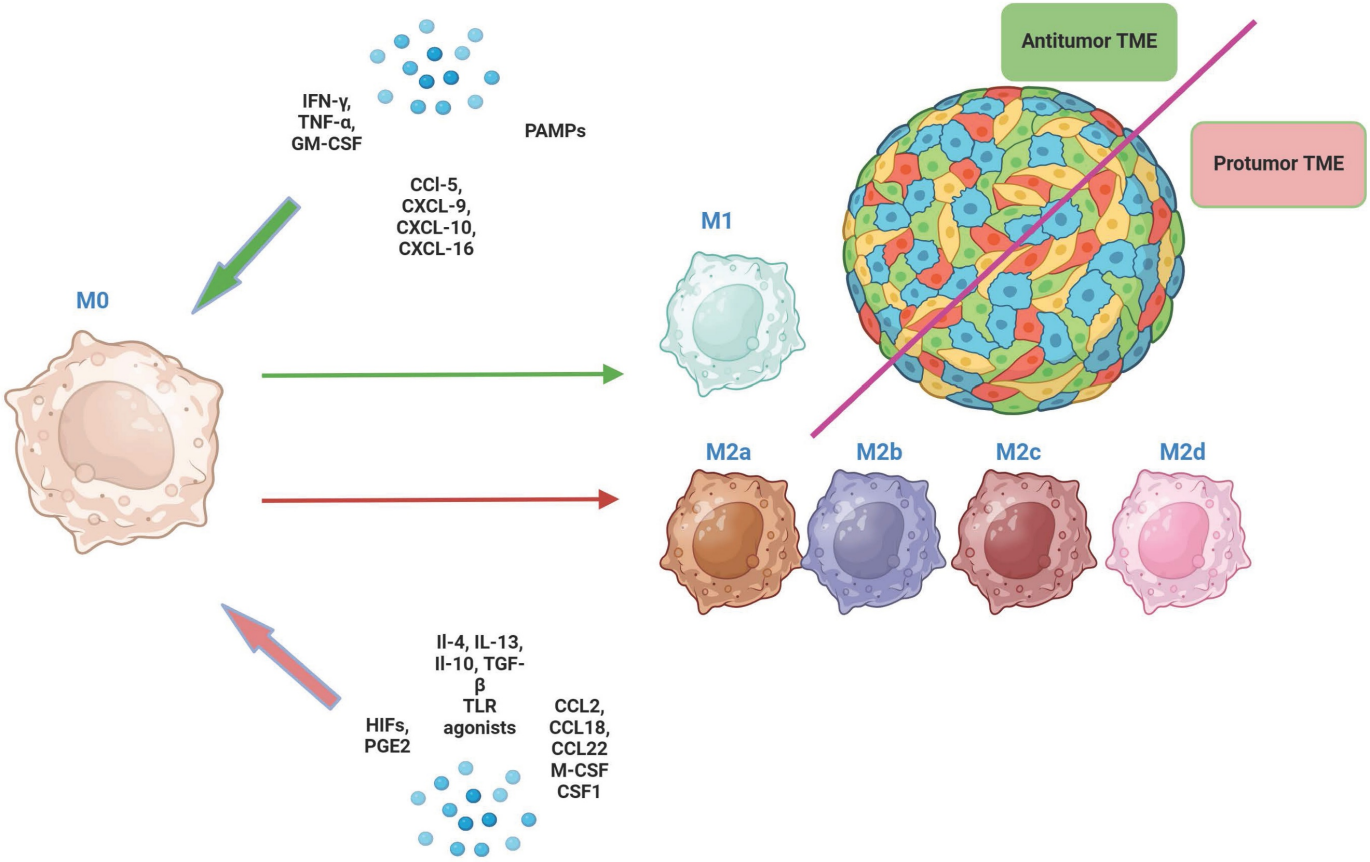
The direction of macrophage differentiation in response to various signals from the TME (the classical paradigm of M1/M2 macrophage dichotomy). Green arrows indicate the direction of macrophage polarization toward M1, and red arrows indicate the direction of polarization toward M2

Macrophages are highly plastic cells, which can acquire different phenotypes in response to signals from TME 7. The 1st and still the most commonly used TAMs classification considers M0 (non-polarized), M1 (classically activated, pro-inflammatory, anti-tumor) and M2 (alternatively activated, anti-inflammatory, pro-tumor) macrophages 7 (Fig. 1).

M1 macrophages are characterized by high phagocytic and antigen-presenting activity and are induced by pro-inflammatory сytokines (IFN-γ, TNF-α, granulocyte-macrophage colony-stimulating factor (GM-CSF), lipopolysaccharide (LPS), chemokines (CCL5, CXCL9, CXCL10, CXCL16) and other factors 11, 14, 15. In turn, they produce a spectrum of pro-inflammatory cytokines (e.g., IFN-γ, IL-12, TNF-α, IL-1β, and IL-6). Through IFN-γ they stimulate T-cell-mediated killing of tumor cells. Additionally, M1 macrophages can recruit and activate other immune cells, including natural killer cells and dendritic cells, bolstering the anti-tumor immune response 16, 17.

M2 macrophages exhibit low phagocytic and antigen-presenting activity and are induced by other cytokines (IL-4, IL-13, IL-10, transforming growth factor-beta (TGF-β), growth factors (macrophage colony-stimulating factor (M-CSF or CSF1), chemokines (CCL2, CCL18, CCL22), prostaglandin E2 (PGE2), or hypoxia-inducible factors (HIFs) 18. They produce matrix metalloproteinases (MMPs) that degrade extracellular matrix and secrete growth factors and cytokines such as VEGF, FGF, IL-6, and IL-12, promoting angiogenesis, tumor growth, invasion, and metastasis. Additionally, M2 macrophages release immune-suppressive cytokines like IL-10 and TGF-β, which can induce fibrosis by activating fibroblasts and promoting collagen production, as well as facilitate epithelial-mesenchymal transition (EMT) 16 ,19. Moreover, by producing proteins like collagen, which shield tumor cells from damage, M2 macrophages can create a favorable environment for tumors to survive and grow 20. Additionally, collagen can interact with integrin receptors on tumor cells, activating signalling pathways that promote cell survival and resistance to death, thereby enhancing viability and proliferation of tumor cells 21.

Counterintuitively, M1 macrophages can also promote tumor progression through activation of NF-κB signalling, production of pro-inflammatory mediators and matrix remodeling 22, 23. M2 macrophages can also have anti-tumor effects by contributing to vascular maturation and “normalization” in CRC, which can limit metastasis and improve prognosis of CRC patients 24-27. These findings suggest that M1 and M2 macrophages may play a more complex role in CRC progression than was previously thought 24, 25.

**Figure 2:**
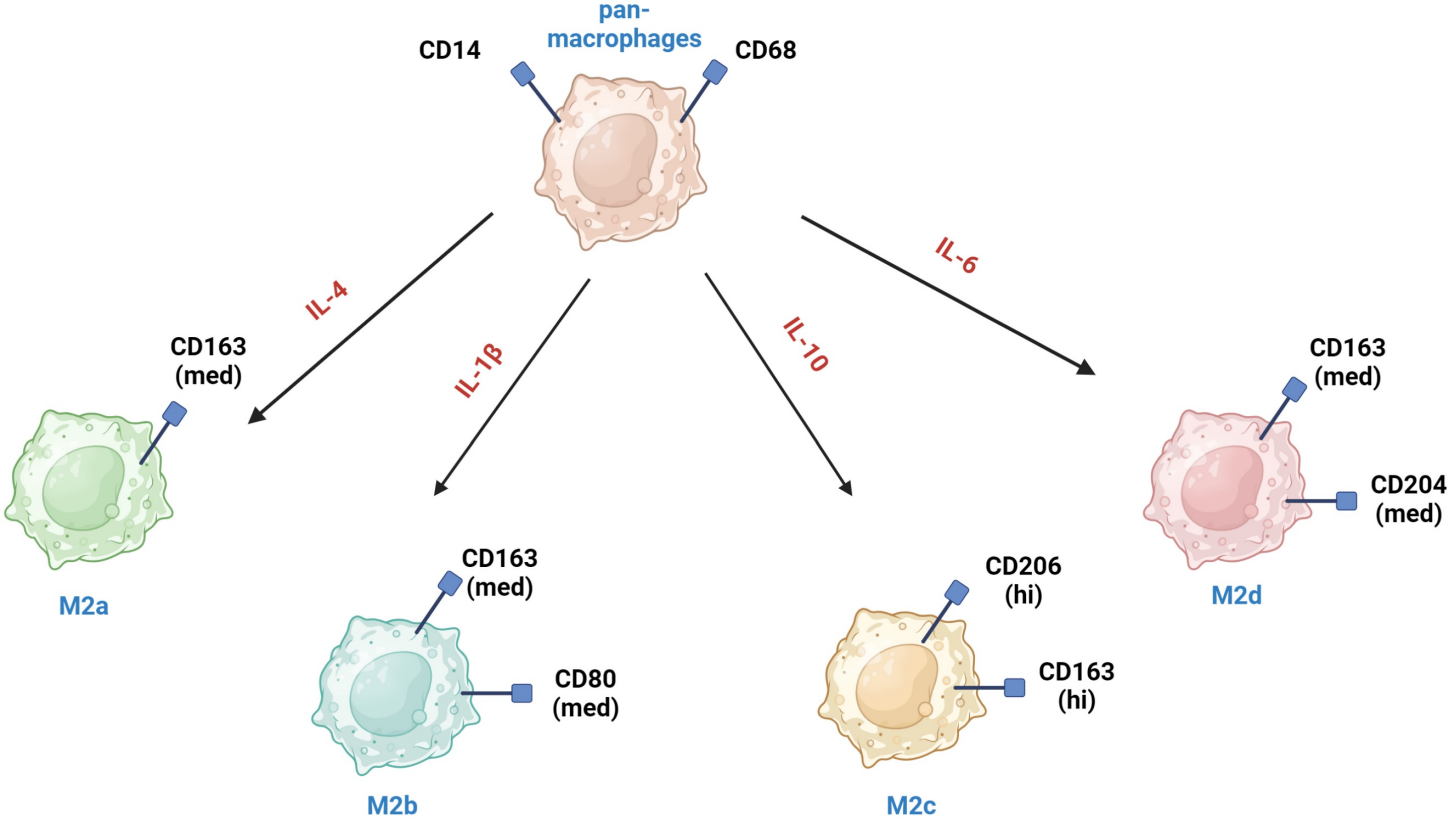
The main phenotypic characteristics of M2a, M2b, M2c, M2d macrophages. In addition to the markers shown in the figure, these macrophages co-express other markers, albeit at lower levels. * med - medium level of expression hi - high level of expression.

M2 macrophages *in vitro* can differentiate into several subtypes (M2a, M2b, M2c and M2d) in response to different signals 26 (Fig. 2). Unique surface marker signatures for each of these subtypes have been identified, enabling further exploration of the potential biological effects of these TAMs 25.

M2a macrophages are induced by IL-4 or IL-13, secrete high levels of IL-4 and IL-13 and express markers such as CD206, CD163, CD209, CLEC7A (CD36), CD204, FCGR3A (CD16a), MSR1, and TIMD4 26, 27. They are involved in tissue repair and remodeling, and they promote tumor growth by enhancing angiogenesis and suppressing the anti-tumor immune response 28. M2b macrophages are induced by immune complexes and Toll-like receptor (TLR) agonists and express high levels of IL-10 and TNF-α. They have a mixed pro- and anti-inflammatory profile and support tumor progression by promoting immunosuppression and facilitating tumor cell invasion and metastasis 29. M2c macrophages are induced by IL-10, TGF-β and glucocorticoids and are characterized by high expression of MARCO, CD206, CD163, CD209 and CD204 30. M2c contribute to tumor progression by promoting an immunosuppressive environment and aiding in tissue repair mechanisms that tumors exploit for growth. M2d macrophages are induced by IL-6 and adenosine and exhibit high levels of VEGF, iNOS, and IL-10. They are strongly associated with angiogenesis and tumor growth 31.

The discovery of several subsets of M2-macrophages has highlighted the oversimplification of the M1/M2 paradigm, since “pure” M1 and M2 forms can only be obtained under *in vitro* conditions 32. *In vivo,* TAMs frequently express a combination of M2 (CD163, CD206), M1 (CD80, CD86, and CD32) and M0 (CD68) markers, reflecting their complexity and plasticity within the TME 11, 27, 33. The majority of TAMs display overlap between M1 and M2 markers, as well as within M2 subtypes, so terms such as “M2-predominant” phenotype appear more appropriate 28, 34.

Supplementary table S1 shows markers that are strongly associated with either the M1 or M2 type as well as others that may be expressed throughout different polarization states. CD68, M0 marker, is expressed by majority of both M1 and M2 TAMs, which probably mirrors polarization process (Supplementary file, Table S1). It is important to note that TAMs can simultaneously co-express M1-associated phenotypic markers (e.g., CD86, CD80, iNOS,) and M2-associated markers (e.g., CD163, CD206, VEGF). This phenotypic plasticity likely highlights the diverse and sometimes contradictory roles these cells play in tumorigenesis and cancer progression 34, 35. Also, co-expression of M1 and M2 markers by a single cell could reflect transitional states between these two types and illustrate significant plasticity of macrophages and dynamic nature of macrophage activation in response to various stimuli from TME. It is hypothesized that TAMs are characterized by a unique phenotype that does not exist under normal physiological conditions and carries both M1 and M2 surface markers 36. Other macrophages, eg. CD169+ (SIGLEC-1) do not fit neatly into the M1 or M2 categories 34, 37-39. CD169+ macrophages were found in various types of cancer in human, as well as mouse cancer models, including CRC with high microsatellite instability 39. In primary CRC, CD169+ macrophages can exhibit pro-tumor effects, whereas in metastasis to regional lymph nodes (RLNs) their effect is mainly antitumor 38.

The above-presented concept of hybrid M1 and M2 phenotypes may explain the contradictory results regarding the biological effects of M1 and M2 macrophage subtypes. This phenotypic ambiguity can lead to mixed immune responses within tumors, potentially influencing cancer progression in unpredictable ways and complicating prognostic assessments 40. Nevertheless, classifying macrophages as M1 or M2 remains relevant for characterizing specific macrophage markers and their biological effects 41.

Given the limitations of the former classification of macrophages, a new one, based on comprehensive scRNA-seq analyses in several human cancers including CRC, has recently been suggested 42 ,43. Based on specific gene signatures and functions, the following types of TAMs have been distinguished: interferon-primed tumor-associated macrophages (IFN-TAMs), immune regulatory tumor-associated macrophages (Reg-TAMs), inflammatory cytokine enriched tumor associated macrophages (Inflam-TAMs), lipid-associated tumor-associated macrophages (LA-TAMs), pro-angiogenic tumor-associated macrophages (Angio-TAMs), RTM-like tumor-associated macrophages (resident-tissue macrophages (RTM-TAMs), glycolytic TAMs (Table 1) 42, 44.

This new classification has improved our understanding of the different types and functions of TAMs beyond the traditional M1/M2 polarization paradigm. Table 1 shows that the biological effects of TAMs are driven by unique combinations of both M1 and M2 markers 45 (Table 1). Notably, TAMs that predominantly exhibit M1 markers often show anti-tumor effects (e.g., angio-TAMs, LA-TAMs), while expression of M2 markers is associated with pro-tumor properties. We suggest that this is due to the unique biological properties of these markers, which are not directly related to the M1/M2 macrophage dichotomy. These observations underscore the need for a more nuanced understanding of the biology of individual macrophage markers, considering their specific functional properties and contextual factors, rather than relying solely on phenotypic classification. In this regard, in our opinion, the classification presented in Table 1, which highlights only 7 types of TAMs, is somewhat simplified, as individual markers can have specific biological roles in cancer development, as well as distinct prognostic and diagnostic significance, as demonstrated in table S1 (Supplementary file, Table S1).

## 3. Macrophages in normal mucosa (NM) of the colon: understanding the distribution and functional implications

During the first three weeks of postnatal life, embryonic macrophages in the colon are replaced by macrophages of monocytic origin, a process linked to microbiota formation. This involves microbiota-induced production of CSF2 and chemokines, which attract circulating monocytes to the intestinal mucosa, where they differentiate into specialized macrophages. This replacement is crucial for developing tolerance to the new microbiota, maintaining intestinal homeostasis, and ensuring appropriate immune responses in the unique gut environment 46.

Resident macrophages can be found in various layers of the colon; however, the largest population of macrophages is observed in the subepithelial portion of lamina propria. They are strategically positioned as the first line of defense against pathogens that occasionally penetrate the epithelial layer 47.

M2 type, which expresses CD163, CD206, CD36 and TREM, and encompasses all their subtypes, represents the majority of resident macrophages in healthy NM 48. Their anti-inflammatory phenotype ensures tolerance to food antigens and commensal microorganisms 49, a process mediated in part by regulatory T cells (Treg) 49. Also, M2 macrophages secrete growth factors that promote proliferation and differentiation of epithelial cells 50 and therefore are involved in repair and remodeling of the mucosa. Besides, M2 macrophages help to regulate the composition of the gut microbiota and prevent dysbiosis 51.

M1 macrophages, although present in smaller numbers, play important roles in maintaining intestinal homeostasis. They monitor potential pathogens using their pattern recognition receptors and present antigens to T cells, contributing to immune surveillance and tolerance to commensal bacteria 52. They are also highly phagocytic, clearing debris and potentially harmful microbes without triggering excessive inflammation. Besides, M1 macrophages participate in maintaining the integrity of the epithelial barrier 52, 53.

Furthermore, NM harbors transitional macrophages between M0 and M2 states, which can be recognized by co-expression of CD68 and CD163 54, 55. Most transitional macrophages located in the subepithelial layer of NM also express CX3CR1, which is crucial for maintaining gut homeostasis by controlling aberrant intestinal inflammation 56. Lack of CX3CR1 leads to increased infiltration by pro-inflammatory macrophages and Th17 lymphocytes in the colon 57.

Macrophages in mucosa-associated lymphoid follicles, which are the most numerous on the border of mucosa and submucosa, help to maintain epithelial barrier function, contribute to immune tolerance to commensal bacteria and promote tissue repair. They predominantly express M2 markers: CD163, CD206 and CD169 58, 59. However, due to high plasticity they can adopt pro-inflammatory (M1-like) phenotype in response to microenvironmental signals 47, 59, 60. Tingible body macrophages (TBM) are found in germinal centers of secondary lymphoid follicles 59. They phagocytize apoptotic B-cells during the germinal center reaction 59. This process, known as efferocytosis, is crucial for maintaining tissue homeostasis and preventing autoimmune responses. Also, TBMs may contribute to the downregulation of germinal center reactions through prostaglandin release, which has been shown to suppress IL-21 production in B cells 59. Intravital imaging has shown that TBMs are stationary cells that use highly dynamic cytoplasmic protrusions to capture and phagocytose migrating fragments of dead cells. The presence of nearby apoptotic cells can trigger the activation and maturation of follicular macrophages into classical TBMs, even in the absence of germinal centers 59.

High densities of M2d macrophages (iNOS+, MSR1+, VEGF+) were identified in the lamina propria, particularly in close proximity to submucosal blood vessels. Under hypoxic conditions, macrophages can activate HIF-dependent signaling pathways that modulate endothelial cell function and promote angiogenesis, thereby supporting vascular remodeling in the lamina propria. Thus, based on previous studies describing hypoxia-responsive macrophage activity and their interactions with stromal and vascular compartments, these cells are likely to contribute to the growth and maintenance of the local microvascular network 61-63.

## 4. Macrophages in colorectal adenoma

Colorectal adenoma (CA) - carcinoma sequence is the most common pathway for CRC initiation. At the early stages of adenomas, M1 macrophages accumulate inside the tumor over M2 macrophages, possibly as a response to the disruption of the epithelial barrier integrity 64. M1 population in adenomas is characterized by expression of CCL3, CCL4, CXCL2 and CCL19, as confirmed by the research of Wierzbicki J. et al 64. As the malignant potential increases from hyperplastic through tubular and tubulo-villous to villous polyps, the expression of CCL3, CCL4, and CCL19 in lesions decreases. Similar dynamics was observed not only in polyps but also in adjacent normal mucosa 65. As the lesion size increased, the expression of CCL3, CCL4, and CCL19 decreased, whereas the expression of CXCL2 increased in the unaffected parts of the colon. 65. Additionally, CXCL2, a chemoattractant for myeloid-derived suppressor cells (MDSCs), suppresses the expansion and activity of anti-tumor effectors, such as T and NK cells 65-67. There are conflicting data regarding CXCL2 expression, with some studies attributing it to M1 macrophages, others to M2 macrophages 68 ,69, and some to both subsets 70.

Macrophages in CA show increased production of MMP9 and MMP2, which are involved in extracellular matrix remodeling and promote the transformation of CA into CRC. MMP+ macrophages share markers of both M1 and M2 types and likely represent transitional cells between these phenotypes 71.

Furthermore, in the case of adenoma progression towards CRC, F4/80-High, MHCII-Low macrophages (characteristic of embryonic tissues and declining postnatally) predominate over F4/80-High, MHCII-High macrophages. F4/80-High, MHCII-Low macrophages consistently demonstrate simultaneous expression of both M1 and M2 macrophage markers 72, 73.

Late-stage adenomas are characterized by predominance of M2 macrophages over M1 type, however, both types of macrophages contribute to creating a favourable pro-tumor microenvironment 74.

CCR2-independent subset of macrophages (predominantly M2c subtype) in CA becomes the dominant resident macrophage population and becomes even more abundant following transformation into tumor. Analysis of CA has shown that CSF1 from neighbour cells within the adenoma microenvironment is a key factor driving macrophage self-renewal 71. Thus, the microenvironment creates an isolated niche for tissue-resident M2 macrophages, which promotes their survival and self-renewal 72, 75.

The transformation of adenoma into CRC is driven by genetic chromosomal instability (CIN), microsatellite instability (MSI), and epigenetic methylation alterations 76. CIN promotes an anti-cancer M1-like macrophage phenotype, enhancing immune responses 77, whereas the IL-1α/IL1R/MyD88/TET2 axis regulates DNA-methyltransferases (DNMTs) such as DNMT1 and DNMT3b in macrophages, thereby directing their polarization toward M2-like phenotype 78. Overexpression of DNMTs in tumor cells downregulates tumor suppressor genes, thereby promoting tumor progression, while M2 macrophages can induce DNMT1 overexpression, further silencing tumor suppressors 79, 80. Progression of late-stage adenoma is marked by dysregulated DNMT methylation and demethylation, shifting macrophages toward an M2 phenotype that enhances production of vascular endothelial growth factor (VEGF) and TGF-β, supporting tumor survival and metastasis 81. Non-coding RNAs, including miR-155 and miR-145, also regulate adenoma-to-CRC transformation 82. miR-155 promotes M1 macrophage polarization, potentially inhibiting CRC progression 83, whereas miR-145 drives M2 polarization by enhancing IL-10 production via HDAC11 targeting and is transferred to macrophages via extracellular vesicles (EVs) 84.

## 5. Macrophages in primary colorectal cancer

The anti-cancer response of macrophages is triggered by the recognition of cancer cells, with TLRs, particularly TLR4, playing a key role by detecting danger-associated molecular patterns released by cancer cells. At this stage, macrophages can phagocytize both apoptotic and viable cancer cells, followed by antigen presentation to T-cells 85. Phagocytosis of apoptotic tumor cells (efferocytosis) is primarily associated with M2 macrophages, which recognize "eat me" signals on apoptotic tumor cells 85. The phagocytosis of viable tumor cells is regulated by a balance between pro-phagocytic "eat me" signals (calreticulin and SLAMF7) and anti-phagocytic "don't eat me" signals (CD47, PD-L1, and β2-microglobulin) on the tumor cell surface 86. CD47 is a key "don't eat me" signal that inhibits phagocytosis by binding to SIRPα on macrophages 87, 88. Blocking CD47 or other anti-phagocytic signals can enhance the clearance of viable tumor cells by macrophages, and is being explored as an anticancer immunotherapy strategy 88, 89. Some viable tumor cells can evade phagocytosis through enhanced expression of these anti-phagocytic signals 87. Phagocytosis of viable tumor cells by macrophages can sometimes lead to the formation of tumor hybrid cells, which may acquire survival advantages and contribute to cancer progression 90.

Majority of TAMs present antigen in the context of MHC II molecules to CD4+ T cells, whereas some macrophages, particularly of the M1 phenotype, can cross-present tumor antigens to CD8+ T cells via MHC class I molecules 91.

### Drivers of macrophage polarization in CRC

The recognition of tumor cells triggers macrophage polarization, which is influenced by both macrophage-intrinsic genetic and epigenetic factors and is extensively regulated by the TME 92. Depending on the molecular cues, these modifications can either suppress or promote CRC progression 93. In CRC, factors such as the IL-1α/IL1R/MyD88/TET2 axis, PAD4, MMP14, MMP9, MMP2, VEGF, HIF-1α, arginase-1, cytokines (IL-4, IL-5, IL-13), and chemokines (CCL2, CCL3, CCL4, CCL5, CXCL12) contribute to macrophage polarization 94, 95. These signals modulate transcriptional activity of macrophages through DNA methylation, histone modifications, and miRNAs induction, playing a critical role in shaping macrophage function in CRC 78, 93.

#### Transcription factors (TFs)

**Figure 3:**
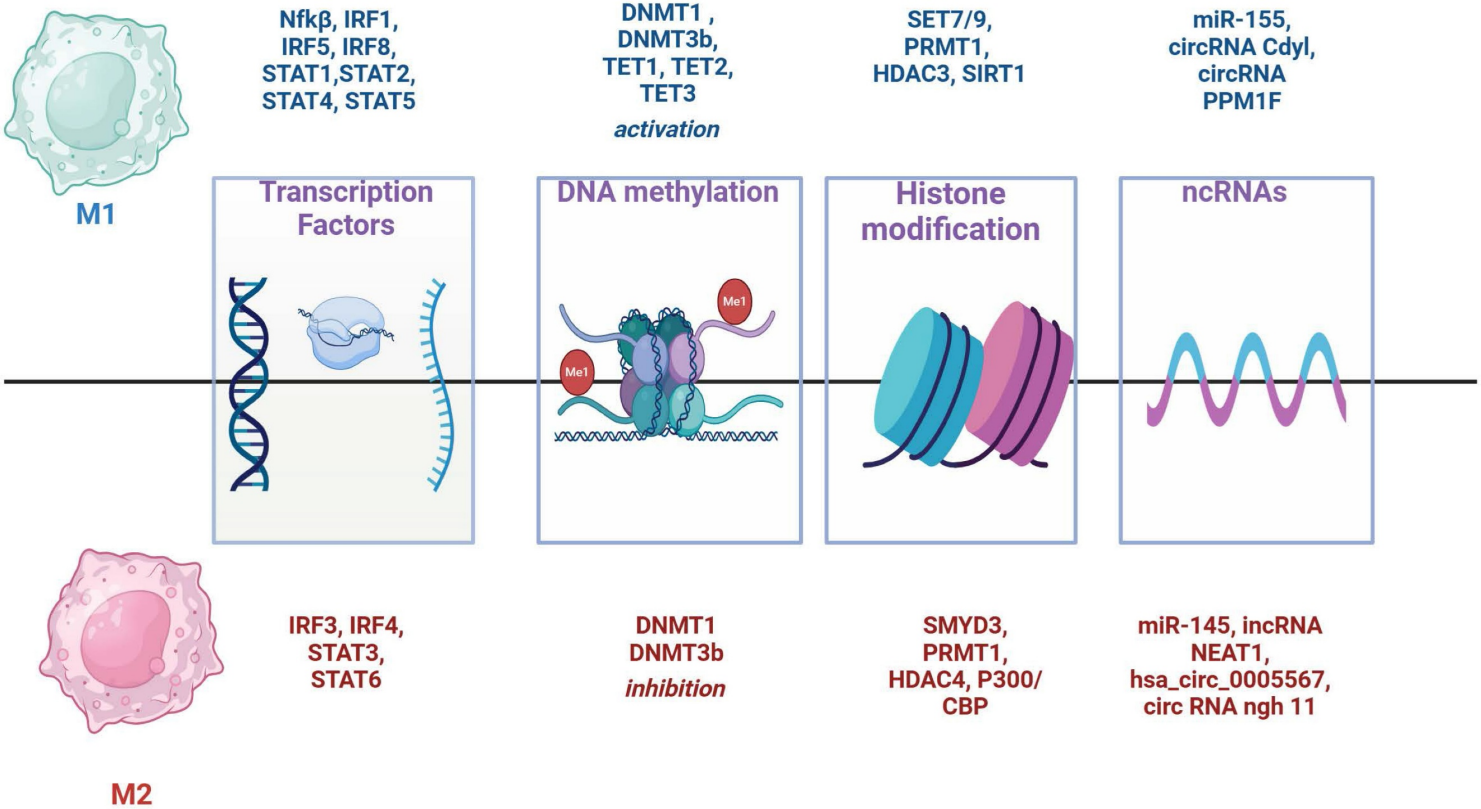
Transcription factors and epigenetic factors determine the direction of M1/M2 polarization of macrophages.

Key transcription factors involved in M1 polarization include IRF1 and IRF5, which mediate the production of inflammatory cytokines TNF-α, IL-6, and IL-12 and also NF-κB and IRF8 (Fig. 3) 96 -98. Members of the STAT-family of TFs (STAT1, STAT2, STAT4 and STAT5) are also important for M1 polarization 99. STAT1 is activated by IFN-γ promoting the expression of pro-inflammatory genes 100. Contrary, IRF-3 and IRF-4 promote M2 polarization 100. Also, STAT3 and STAT6 proteins are central to M2 polarization. STAT6 is activated by IL-4/IL-13 promoting the expression of M2 markers such as the mannose receptor and PPARγ. IRF4 competes with IRF5 and upregulates STAT6 102, 103.

#### DNA methylation and demethylation

Two types of DNA methyltransferases (DNMTs), enzymes responsible for DNA methylation, promote macrophage polarization toward the M1 type: DNMT1 and DNMT3b (Figure 3). DNMT1 plays a crucial role in M1 activation by suppressing the expression of Krüppel-like factor 4 (KLF4) and suppressor of cytokine signaling 1 (SOCS1), thereby enhancing the secretion of proinflammatory cytokines such as TNFα and IL-6 79, 104. DNMT3b targets and inhibits the PPARγ promoter, a positive regulator of M2 macrophage polarization 105, and knockdown of DNMT3b can promote M2 polarization 80.

Demethylation is equally important and is controlled by ten-eleven translocation (TET) enzymes, specifically TET1, TET2, and TET3, which oxidize 5-methylcytosine (5mC) to 5-hydroxymethylcytosine (5hmC), ultimately removing the methyl group 106. This demethylation favors M2 macrophage polarization 107. The balance between DNMT and TET activity is crucial for maintaining macrophage M1/M2 plasticity 108. DNMT inhibition may shift macrophages toward the M2 phenotype, potentially promoting an immunosuppressive environment that supports tumor growth 109. Conversely, demethylation can also activate pro-inflammatory genes, promoting M1 polarization in response to inflammatory signals. In M1 macrophages, increased TET activity can drive the expression of key cytokines and inflammatory markers, contributing to their anti-tumor function. DNMT inhibitors, such as azacitidine and decitabine, promote demethylation and reactivation of silenced genes making them useful in anticancer therapy 110. These inhibitors could also enhance the M1 macrophage response, potentially improving the efficacy of immune-based cancer treatments 80.

#### Histone modifications

Histones undergo post-translational modifications such as methylation, acetylation, phosphorylation and ubiquitination. These modifications can influence chromatin structure thereby affecting gene expression and altering the accessibility of DNA sites to transcription factors. Histone methyltransferases (HMTs) play a specific role in macrophage polarization (Fig. 3). For example, SET7/9 activates NF-κB, promoting M1 polarization by inducing TNF-α production, whereas SMYD2 inhibits M1 polarization by regulating pro-inflammatory cytokines 108. In contrast, SMYD3 promotes M2 polarization by activating pathways that drive M2 macrophage phenotypes 111. PRMT1, an arginine methyltransferase, is involved in both M1 and M2 polarization through distinct mechanisms 108. Histone acetyltransferases (HATs), such as P300/CBP, and histone deacetylases (HDACs) also influence macrophage polarization 112. P300/CBP is crucial for M2 macrophage polarization 112, while HDAC3 supports M1 activation 113.

#### ncRNA

Non-coding RNAs (ncRNAs) can regulate gene expression and maintain the balance between M1 and M2 macrophages by modulating transcription, translation and mRNA splicing. miR-155 and miR-145 are particularly important in directing M1/M2 macrophage polarization 114, 115 (Fig. 3). Various other ncRNAs, including circRNAs and lncRNAs, also play significant roles in macrophage polarization by influencing key signaling pathways such as NF-κB and the miR-224-5p/IL-33 axis. These regulatory networks contribute to the complex dynamics of macrophage polarization within the tumor microenvironment 108, 116.

Epigenetic regulation is crucial for shaping macrophage function within the TME, influencing their polarization and secretory profiles. Key targets include extracellular matrix (ECM)-modifying enzymes, such as MMPs and ADAM family proteases. ncRNAs such as miRNAs, circRNAs, and lncRNAs modulate the expression of genes involved in ECM remodeling by directly or indirectly regulating MMP activity 117.

Through these epigenetic mechanisms, macrophages can fine-tune the expression of proteolytic enzymes, thereby influencing tumor invasion, metastasis, and immune evasion. Interestingly, the numbers of MMP+ and ADAM8+ TAMs in CRC are correlated, with both populations secreting disintegrin and metalloprotease domain 8 (ADAM8). ADAM8 activates MMPs, driving matrix remodeling and promoting tumor invasion. Additionally, ADAM8 protein is involved in cell migration, adhesion, and membrane shedding, processes that are critical for metastatic spread. Inhibition of ADAM8 and MMP activity impedes the invasive and migratory capabilities of drug-resistant CRC cells, suggesting that ADAM8 may serve as a macrophage-related biomarker in CRC, warranting further investigation 118.

IL-10 and TGF-β contribute to an immunosuppressive environment by inhibiting cytotoxic T cells, enhancing the recruitment of MDSCs and promoting regulatory B cells 119. M1 TAMs, although less abundant, primarily exhibit anti-tumor effects and are associated with favorable prognosis in CRC 120, 121.

The presence of mixed M1/M2 macrophages (particularly CD68+, CD80+, MHC-II+) in the TME correlates with reduced frequencies of liver metastasis 122, 123, and high infiltration of CD68+ TAMs is considered a favorable prognostic marker in CRC 124.

In the early stages of CRC, M1 macrophages cooperate with M2 macrophages to promote angiogenesis through secretion of VEGF and iNOS. Subsequently, the density of CD206+, CD163+ M2 macrophages, which secrete PDGF-B and high levels of MMP-9, gradually increases. This is necessary for the maturation of growing blood vessels and remodeling of the vascular network. PDGF-B facilitates the recruitment of pericytes, which contribute to stabilization of sprouts and their evolution into functional vessels 125, 126. Overall, increased angiogenesis in tumors is often associated with poor prognosis, contributing to CRC progression and metastasis. Several studies have identified CD206 as a potential biomarker associated with poor prognosis in CRC 125-127. However, other report that a high density of certain CD206+ and CD163+ M2 TAMs positively correlate with a prolonged relapse-free interval in CRC patients. They are also associated with lower tumor aggressiveness and fewer lymph node metastases 40, 126. Similar trends have been observed in experimental CRC growth models and other cancer types 129. For instance, in syngeneic mouse tumor models, increased CD206+ TAM were associated with reduced tumor burden, and in patients with cutaneous melanoma, high CD206+ macrophages density correlated with improved overall survival. This phenomenon is attributed to "vascular normalization", whereby stabilized and mature vessels prevent tumor cells from entering the vascular network 130, 131, which helps explain conflicting data regarding the prognostic role of M2 TAMs in CRC.

Recent studies have identified a subset of secreted phosphoprotein 1 (SPP1), also known as osteopontin positive macrophages within the tumor tissue that possess unique characteristics and immunosuppressive properties 131. SPP1 is expressed by both the M2- and M1-macrophages. SPP1+ TAMs are primarily located at the invasive front of the tumor in close proximity to both CRC cells and fibroblasts, where they contribute to EMT, angiogenesis, tumor growth, invasion and metastasis 131, 132. Targeted downregulation of SPP1+ in TAMs has been investigated as a potential therapeutic strategy 133. SPP1+ TAMs differentiate from THBS1+ TAMs, which are also associated with poor prognosis in CRC` and typically exhibit an M2 phenotype. THBS1 is highly expressed in the stromal areas of both primary and metastatic CRC lesions and contributes to exhaustion of cytotoxic T-cell and impaired vascularization, correlating with the aggressiveness of the disease 131.

These findings indicate that single markers are insufficient to differentiate TAM phenotypes, which often possess antagonistic functions (Table 2). Table 2 demonstrates the cross-expression of M1 and M2a-d macrophage markers. Multiplex staining with a panel of markers is recommended to accurately characterize TAMs 128. Wang et al., 2023 have recently demonstrated that markers of M1 macrophages (NOS2, CXCL10, CD11c) were weakly expressed in both the invasive front and tumor center (TC), whereas M2 markers (CD163, CD206, CD115) were primarily expressed in the invasive front in primary CRC. CRC patients with low M1 markers expression or high M2 markers expression demonstrated poor prognosis. Importantly, the combined prognostic value of a multiple markers (NOS2/CXCL10/CD11c or CD163/CD206/СD115) was higher than that of any single marker 128.

### Macrophages and tumor microarchitecture

Beyond the complexity of their various polarization states, macrophages located in different regions of CRC can also play opposing roles 40, 94, 134. The inherent plasticity of TAMs enables dynamic transitions between M1 and M2 types in response to microenvironmental signals. In the TC, which is characterized by a high degree of genetic instability, cellular proliferation and hypoxia, TAMs are predominantly polarized toward an M2-like type 134. The ratio of M1 to M2 macrophages may influence the progression and metastatic potential of CRC and is considered an important prognostic marker 94.

The tumor invasive margin (TIM) is the area where cancer cells actively invade the surrounding tissue, exhibiting a more aggressive and invasive phenotype. Pinto et al. reported a predominance of CD163+ TAMs in the invasive front, whereas CD80+ TAMs were enriched in intratumoral region (IT) and the invasive front 135. Compared to TAMs in the tumor core, those in the TIM often display a more complex and mixed (M0, M1 and M2) phenotype, which reflect their adaptability to the dynamic tumor-host interface 136.

Several studies have demonstrated correlation between the types and abundance of TAMs in invasive front with the clinical and pathological features of CRC. For example, the number of M2 TAMs, predominantly CD163+, increases in the TIM as tumor advances. High numbers of M2 TAMs at TIM are associated with poor histological differentiation, lymphovascular invasion and lymph node metastasis. Additionally, it was found that a higher M1/M2 ratio in the TME, particularly at the invasive front, correlates with a better prognosis and lower risk of metastatis 137, 138. The inner and outer layers of TIM exhibit distinct tissue architecture and different immune landscapes 139. Moreover, individual immune cell types, including different subsets of T cells, NK cells, dendritic cells (DCs) and macrophages, can show different prognostic associations depending on whether they are located in the inner or outer layers of TIM, as demonstrated by Artur Mezheyeuski et al 140, 141. Tumor stage stratification revealed that TAMs, especially CD163+, were more abundant in TIM of T3 tumors, whereas CD80+ macrophages predominated in less invasive T1 tumors. In advanced (T3-T4) CRC a higher CD68+/CD163+ cell ratio and a lower CD80+/CD163+ cell ratio were associated with shorter overall survival 135.

The peritumor zone (PT) lies adjacent to the invasive margin. Determined on the depth of the invasion, the PT region may include the submucosa, muscularis propria and subserosal fat. Immune cells in this region play a crucial role in tumor progression, invasion, and metastasis, as well as in shaping the local immune response against the tumor. Tertiary lymphoid structures, which are enriched in CD68+ macrophages are frequently seen within the PT 142. TAMs in the PT can interact with fibroblasts, endothelial cells, and other immune cells to modulate angiogenesis, immune responses, and support stromal expansion 143. TAMs in PT area can co-express a range of both M1 and M2 markers, including CD68, CD163, and CD206, reflecting their involvement in both pro- and anti-inflammatory activity 136, 144.

In summary, TAMs in TC and PT predominantly exhibit an M2-like phenotype that promotes tumor progression and may enhance immunosuppression or support survival of tumor cells under hypoxic conditions. TAMs in invasive front demonstrate a more complex phenotype capable of supporting both tumor growth and anti-tumor immunity, which is crucial for invasion and metastasis of tumor cells 135, 145.

Conflicting findings regarding the predominant TAMs phenotypes in CRC can be explained through the perspective of CMS, which are based on differential gene expression within the TME. The four recognized CMS groups, CMS1 (MSI-immune), CMS2 (canonical), CMS3 (metabolic), and CMS4 (mesenchymal), are distinguished by specific genetic alterations and intratumoral immune profiles 146, 147. CMS1 is characterized by microsatellite instability followed by immune activation and dense infiltration by cytotoxic and memory T-cells, Th1 T cells, follicular helper T-cells, γδ T-cells, as well as activated DCs and NK cells 146, 147. Macrophages in this subtype are predominantly polarized towards the M1 phenotype 145. CMS2 tumors show activation of WNT and MYC pathways, while CMS3 tumors exhibit metabolic dysregulation and frequent KRAS mutations. Mixed M1/M2 macrophage phenotypes are common in CMS2 and CMS3, and the balance between pro- and anti-tumor macrophage effects depends on the M1/M2 ratio as well as the metabolic context 145. CMS4 tumors are defined by EMT, associated with matrix remodeling, high stromal activity, TGF-β pathway activation, and angiogenesis. Compared with CMS1 tumors, CMS4 lesions contain fewer CD8⁺ and CD4⁺ T cells but more regulatory T cells, monocytes, eosinophils, myeloid cells, and resting DCs. Macrophages in CMS4 predominantly exhibit an M2 phenotype, which contributes to a strong pro-tumor microenvironment 148. It is also important to consider the existence of mixed CRC subtypes and the potential for tumors to transition between CMS categories. Such heterogeneity complicates the assessment of M1/M2 dominance and its prognostic implications. Therefore, evaluating the biological role of individual macrophage markers and their influence on tumor progression or regression remains highly relevant 148.

## 6. Role of macrophages in regional lymph node (RLN) metastases

RLN metastasis occurs in several stages: (1) initiation of lymphangiogenesis in the primary tumor, (2) migration of the first tumor cells together with immune cells through lymphatic channels toward the draining lymph nodes, (3) induction of lymphangiogenesis within the RLN, (4) formation of a pre-metastatic niche in the RLN, and (5) proliferation of tumor cells in the RLN parenchyma. At each of these stages, macrophages play a specific role in regulating, initiating, or suppressing these processes 149, 150.

The role of M2 macrophages (particularly the M2d subtype) in the production of VEGF-C, which drives lymphangiogenesis, is well established 151. VEGF-C also engages its lymphatic endothelial targets, leading to reduced expression of vascular endothelial cadherin (CD144) and disruption of the endothelial barrier, thereby facilitating tumor cell entry into lymphatic vessels 152. A high number of M2 macrophages and a low number of M1 at the tumor invasive front correlate with lymphovascular invasion and poor histological differentiation 141. Furthermore, the M2/M1 ratio is a stronger predictor of RLN metastasis than the absolute number of pan-, M1, or M2 macrophages at the invasive front. These findings suggest that M2 TAMs at the invasive front may contribute to CRC progression from stage II to stage III 141, 152.

Hydrodynamics plays a key role in the spread of tumor cells into RLNs. Blood vessels in the tumor typically exhibit abnormal permeability and disrupted blood flow, causing plasma to accumulate in the extracellular spaces and impairing drainage due to compression of local lymphatics 153, 154. As a result, intratumoral interstitial fluid pressure (IFP) increases, generating an IFP gradient that facilitates tumor cell migration toward the RLN 154. Interstitial flow has been shown to polarize macrophages towards an M2-like phenotype (CD163+, CD206+) through integrin/Src-mediated mechanotransduction pathways involving STAT3/6 155. Consistent with this flow-induced polarization, M2 macrophages demonstrate a higher migratory capacity. Interstitial flow also recruits M2 macrophages to tumor masses, where they promote cancer cell invasion through the secretion of MMPs and growth factors, such as TGF-β, which degrade the ECM and support the invasive and metastatic potential of cancer cells. Additionally, macrophages release immunosuppressive cytokines (e.g., IL-10), which suppress anti-tumor immune responses, further contributing to tumor progression 152, 155.

Macrophages, mainly of the M2 subtype, are naturally present in mesenteric lymph nodes 156. Additionally, M2 macrophages infiltrate non-metastatic RLNs even before metastatic spread occurs 157, 158. These M2 macrophages serve as a source of angiogenic factors (VEGF-C, iNOS), which induce lymphangiogenesis and angiogenesis in the RLNs, thereby multiplying potential routes for metastatic dissemination 141. M2 macrophages also contribute to the formation of the premetastatic niche in RLN by remodeling of ECM via secreted MMP-9 159. In addition, M2 macrophages create an immunosuppressive environment in lymph nodes before metastasis occurs, secreting cytokines such as IL-10 and recruiting regulatory T cells (CCR-6+ Tregs) to the developing premetastatic niche 160. A study by Yanping Wang et al., 2021, demonstrated elevated numbers of M2b, M2c, and M2d macrophages (CD163+, CD206+, VEGF+, iNOS+) in exposed RLN. Moreover, RLNs with macrometastases contained significantly higher numbers of M2 macrophages than those with micrometastases 152.

Anti-tumor effects of CD169+ macrophages within RLNs have also been demonstrated. The density of CD169+ macrophages in RLN correlates positively with the density of infiltrating T- or NK-cells in tumor tissues, indicating the significance of CD169+ macrophages in anti-tumor immune responses 161.

## 7. Role of macrophages in distant hematogenous metastases

Macrophages can modulate distant CRC metastasis at every sequential stage of the metastatic process: invasion of tumor cells, angiogenesis in primary CRC (pCRC), intravasation and survival of tumor cells in the circulatory system, formation of pre-metastatic niches in distant organs, extravasation of tumor cells, colonization leading to micro-metastases formation, and proliferation of tumor cells at the secondary sites 162.

### Invasion of CRC cells

During EMT, epithelial-like, early proliferating cancer cells lose intercellular adhesions and acquire a fibroblast-like phenotype with enhanced invasive and migratory properties, which is a prerequisite for metastasis 163. EMT can also confer stem cell-like characteristics on cancer cells, enhancing their ability to initiate new tumors and resist therapies 164.

M2-macrophages, through secretion of MMPs, TGF-β and IL-8, play the key role in driving EMT 94, 165. However, M1 macrophages are also required, as a source of IL-6, TNF, and IL-1 166. Cytokines produced by M1 and M2 macrophages can act synergistically to create a pro-EMT environment. For example, TNF-α (from M1) and TGF-β (from M2) can cooperate to enhance EMT and stem cell-like properties in CRC cells through the NF-κB/Twist axis 167, 168.

### Angiogenesis in primary tumor

Angiogenesis plays a crucial role in supplying nutrients and oxygen to support tumor growth and progression. M2a and M2d macrophages (iNOS+, VEGF+ CD204+, CD163+) can secrete pro-angiogenic factors that promote the formation, maturation and stabilization of new blood vessels in within the primary tumor 68.

M1 macrophages also contribute to angiogenesis through the secretion of VEGF-A and FGF2, both essential for capillary sprouting 169. For instance, in a single-cell atlas of tumor-infiltrating myeloid cells, a cluster of TAMs with an M1-like phenotype coexists with a macrophage subpopulation exhibiting strong angiogenic properties, which was associated with poor prognosis 170, 171. Several studies have shown the joint contribution of M1 and M2 macrophages to angiogenesis and tumor progression 126, 172. Contrary, Bi Y. et al. 2020 reported that M1 macrophages may inhibit angiogenesis and tumor growth by promoting the production of CXCL9, CXCL10, and CXCL11 in CRC, and that their predominance may serve as a marker of favorable prognosis in CRC 173.

### Intravasation, circulation and extravasation of cancer cells

Intravasation and extravasation are pivotal stages of the metastatic cascade. M2 macrophages play a leading role in these processes, with smaller contribution from M1 and transitional M1/M2 macrophages 133, 174. During intravasation, tumor cells detach from the primary tumor and enter blood vessels. MMPs remodel the stromal ECM and degrade the basement membrane, making the tumor stroma and endothelial barrier more permissive to the intravasation of CRC cells 175. Epidermal growth factor (EGF), predominantly secreted by CD206+ TAMs, promotes the invasion and mobility of CRC cells, and activation of EGFR on tumor cells is required for sustained intravasation 176. VEGF produced by M2-like TAMs increases vascular permeability, facilitating both intravasation and extravasation 177.

Importantly, TAMs can directly "guide" cancer cells to blood vessels (migratory macrophages) and assist their entry into the bloodstream (sessile perivascular macrophages) 177. The following sequence of events was previously described: TGF-β, produced by tumor tissue, induces expression of CXCR4 by TAMs. At this stage, CXCR4⁺ TAMs then interact with cancer cells and induce expression of actin regulators, ultimately leading to the formation of podosomes in TAMs and invadopodia in tumor cells. These specialized structures facilitate extracellular matrix degradation and enhance metastatic spread. Such mechanisms have been documented in lung, ovarian, breast, and prostate cancer metastases. 178, 179. Additionally, chemokine CXCL12 produced by perivascular fibroblasts can attract CXCR4+ TAMs together with mobile cancer cells toward blood vessels. After tumor cells enter the circulation, migratory TAMs differentiate into perivascular macrophages (primarily M2), increasing vascular permeability and supporting tumor-cell intravasation 179, 180.

Macrophages may also promote CRC cell survival in the bloodstream by releasing cytokines and chemokines. For example, IL-6 from both M1 and M2 macrophages activates the JAK-STAT3 pathway, supporting tumor-cell survival and proliferation 181.

In the liver, M2-type macrophages expressing CD163 and CD206 produce hepatocyte growth factor (HGF), which engages the c-Met tyrosine kinase receptor on the surface of migrating tumor cells, promoting their extravasation into the liver through activation of various signalling pathways, including JAK/STAT3, MAPK, PI3K/AKT, and NF-κB 181- 183.

### The premetastatic niche (PMN) in the liver

In CRC, the liver is the most common site of distant metastases. The development of the hepatic PMN and the imprinting of macrophages within this niche depend on tumor-derived EVs, which circulate systemically. Integrins on EV surfaces mediate organotropism, with specific integrin patterns correlating with metastatic destination 184. EVs carry RNA, lipids, metabolites, and proteins that reprogram recipient cells, modulate immunity, remodel the ECM, and promote angiogenesis. In the liver, tumor-derived exosomes containing miR-934 and miR-135a-5p bind preferentially to a subpopulation of CD206+ resident Kupffer cells (KC) 184-187. This promotes upregulation of the fatty acid transporter CD36 and polarizes KCs toward an anti-inflammatory M2 phenotype, contributing to PMN formation through PTEN suppression and PI3K/AKT pathway 188, 189.

KCs are present in both normal liver tissue and the metastatic TME, although their phenotypes differ (Supplementary file Table S2). These differences are not absolute, and KCs populations remain heterogeneous. Their specific roles may vary depending on metastasis stage, interactions with other immune cells, and microenvironmental cues 190, 191.

The dominant macrophage populations in CRC liver metastases (LM) are M2 macrophages (CD206+, CD163+) and KCs, although pan-macrophages (CD68+), M1 macrophages (CD86+), and transitional forms are also present 174, 187. High M2 macrophages abundance correlates with poor prognosis 192 (Supplementary file, Table S2).

M2 macrophages are pivotal in recruiting MDSCs to the liver, which is a key step in PMN formation. CRC cell-derived VEGF-A triggers M2 macrophages (especially CD163+ and CD206+) to produce CXCL1, which attracts CXCR2+MDSCs to the pre-metastatic site, promoting liver metastasis 193, 194.

Macrophages may also contribute to PMN formation through fusion with tumor cells, forming hybrids that promote distant metastases. In mouse studies, macrophage-melanoma hybrids injected into mice produced pancreatic metastases 195. These hybrids may aid PMN formation, though further research is needed to clarify their role in extravasation and colonization.

### Colonization of liver by tumor cells to establish micro-metastases

On human breast cancer cell lines (MDA-MB-435, MDA-MB-231, T47D, and MCF7), stimulation of the CCL2/CCR2 axis in monocytes and macrophages increased CCL3 gene transcription and protein production, which in turn enhanced macrophage retention at metastatic sites 196. Moreover, in CRC LM, the CCL3/CCR1 axis mediates direct macrophage-tumor cell interactions, partly via an α4 integrin, while CCR2+ M2 macrophages drive metastatic progression; accordingly, CCR2 mediated regulation of CCL3 is a potential therapeutic target 197.

CCR2+ macrophages at the metastatic site support further metastases by increasing vascular permeability (via VEGF-A), providing survival signals to metastatic tumor cells, and contributing to immunosuppression within the metastatic niche 198, 199. Additionally, as noted above, cancer-associated fibroblasts (CAFs) further enhance metastatic potential by recruiting monocytes through IL-8/CXCR2 signaling and promoting M2 polarization through IL-6-mediated VCAM-1 upregulation in tumor cells. In metastasis, CAFs and macrophages interact, increasing the metastatic potential and supporting the colonization and outgrowth of disseminated tumor cells 174, 200.

### Proliferation of tumor cells at the LM

After tumor cells colonize the liver, macrophages, particularly M2 ones, continue to support metastatic growth. In CRC LM, M2 macrophages (whether free or physically associated with tumor cells) secrete HGF, which activates c-MET and downstream MAPK, ERK1/2, and RAS pathways, driving tumor-cell proliferation 201, 202. Simultaneously, M2 macrophages are recruited into the LM through binding of the matricellular protein SPON2. The expression of SPON2 positively correlates with increased M2-TAM infiltration and poor prognosis in CRC. Furthermore, SPON2 promotes cytoskeletal remodeling and transendothelial migration of monocytes via the integrin β1/PYK2 axis. It may also indirectly promote M2 polarization by increasing IL-10, CCL2, and CSF1 expression in tumor cells. Blocking M2 polarization or depleting macrophages suppresses SPON2-induced tumor growth and invasion, while inhibition of the SPON2/integrin β1/PYK2 axis reduces transendothelial monocyte migration and TAM-mediated cancer progression 203. When assessing macrophage phenotypes in metastasis, it is essential to consider the CMS subtypes. Metastatic CMS subtypes can differ from those of the primary CRC 204. Metastatic propensity is highest in CMS4 (mesenchymal) primary tumors and lower in CMS1 and CMS3. Approximately 90% of liver metastases belong to one of two subtypes, either CMS2 (canonical) or CMS4 (mesenchymal).

**Figure 4:**
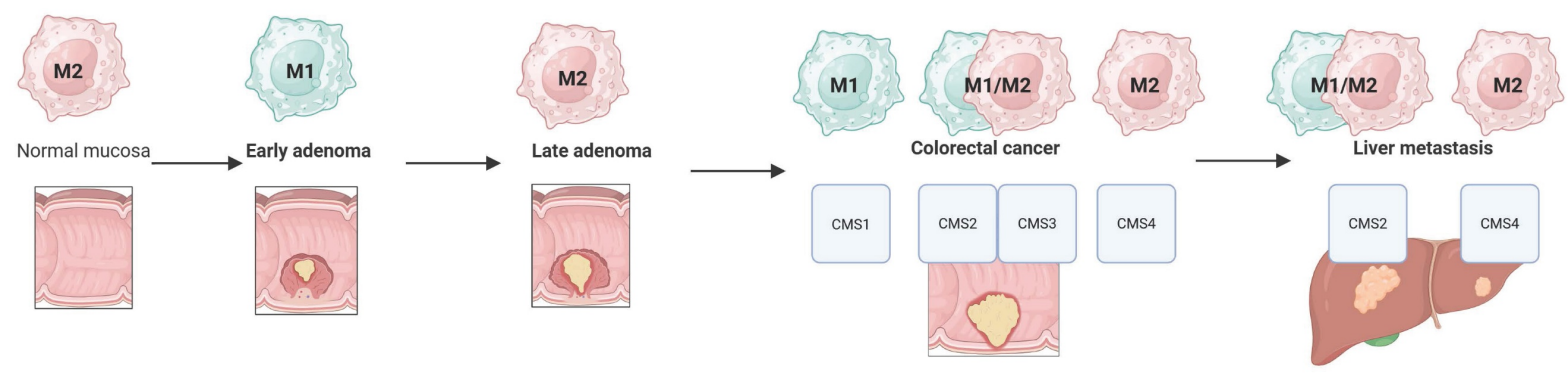
Macrophage M1/M2 phenotypes in the adenoma-colorectal cancer-liver metastasis sequence.

Figure 4 summarizes dominant macrophage phenotypes across the adenoma-colorectal cancer-liver metastasis sequence, considering CMS subtypes. Across this progression macrophage populations are highly heterogeneous, with numerous mixed and transitional forms that influence biological activity and prognosis 204.

## Conclusions

Despite the diversity of macrophage phenotypes, including their mixed forms observed in both adenoma and colorectal cancer, as well as its liver metastases, the following phenotypic portrait of macrophages can be outlined in the sequence: normal mucosa (M2) - early adenoma (M1) - late adenoma (M2) - colorectal cancer and liver metastases (the predominance of M1/M2 is determined by CMS subtypes) (Fig. 4).

Macrophages play a complex and multifaceted role in CRC initiation, progression, and metastasis. Their plasticity enables them to exert both pro- and anti-tumor effects depending on the specific TME and stage of disease. The traditional M1/M2 paradigm is oversimplified, as macrophages in the TME often display mixed phenotypes. Also, macrophage phenotypes and functions evolve through CRC progression from normal mucosa through adenoma to invasive carcinoma and metastasis. The spatial distribution of macrophages within tumors (e.g. tumor center vs invasive margin) impacts their functional roles and prognostic significance. Macrophages are active players in the metastatic cascade, including the stimulation of angiogenesis, intravasation, and extravasation of tumor cells, as well as the creation of pre-metastatic niches.

The complexity and diversity of macrophages in liver metastasis underscore their pivotal roles in cancer progression and highlight the potential for macrophage-targeted therapies to improve patient outcomes 205

Given the heterogeneity of TAMs, advanced techniques that enable spatial visualization of distinct macrophage phenotypes are needed. Currently, one of the most promising methods is spatial transcriptomics (ST) 131, 206. ST allows simultaneous measurement of thousands of genes while maintaining spatial context and enables unbiased discovery of novel macrophage subtypes. It also provides insights into cell-cell interactions, and reveals functional states of macrophages in different tumor regions. Integration of ST with other -omics data and advanced computational methods will be crucial to fully elucidate macrophage biology in colorectal cancer and develop more effective targeted immunotherapies.

Despite considerable research on macrophage involvement in CRC, several gaps and inconsistencies remain unresolved. First, the prognostic significance of M2 macrophages continues to spark the debate. While numerous studies associate elevated densities of CD163+ or CD206+ cells with adverse patient outcomes, other studies showed that M2 macrophages can promote vessel “maturation” and potentially improve prognosis by contributing to vascular normalization. Such divergence likely arose from the fact that M2 cells encompass multiple subtypes (e.g., M2a, M2b, M2c, M2d) and often exhibit mixed phenotypes with overlapping M1/M2 markers *in vivo*. Therefore, future research should focus on expanding standard immunohistochemical panels beyond CD163/CD206 and establishing uniform criteria for subclassifying macrophage populations both at the protein and transcriptome levels.

Second, conflicting data persist regarding the role of M1 macrophages. Traditionally viewed as anti-tumor effectors, M1 cells can also foster chronic inflammation, promote EMT, and facilitate tumor dissemination. This “dual face” phenomenon may result from contextual factors such as region (tumor center vs. tumor invasive margin vs. regional lymph nodes) and the specific molecular subtype of CRC (CMS1 through CMS4). Future work must incorporate molecular subtyping and spatial transcriptomics to clarify how distinct TAM niches interact with host tissue, potentially guiding novel prognostic tools and targeted therapeutic strategies.

Another challenge lies in reconciling genetic and epigenetic determinants of macrophage polarization. Whilst some data suggest that DNMT1/3b suppression skews cells toward an M2-type, other models associate these enzymes with fostering M1-related inflammatory cascades. Elucidating the full scope of DNA methylation, histone modifications, and non-coding RNA regulation of TAM subsets will require large studies in patient-specific organoids and *in vivo* models, integrating methylome, transcriptome, and proteome analyses of both tumor and its microenvironment.

Lastly, the translational potential of reprogramming macrophages remains unexplored in CRC. Therapeutic approaches aimed at blocking “don't eat me” signals (e.g., CD47, PD-L1) or at converting M2 to M1 phenotypes *in situ* are yet to be systematically evaluated in the context of different TAM subtypes. In conclusion, combining multi-marker phenotyping, spatial biology, and advanced epigenetic profiling offers a promising route to resolve current controversies, refine patient stratification, and pave the way to more precise therapies that harness the plasticity of macrophages to combat colorectal cancer.

## Supplementary Material

Supplementary table.

## Figures and Tables

**Table 1:** Classification units of TAMs based on single-cell sequencing data.

Types of TAMs	Protein markers	Cytokines	Functions/Effects
Interferon-primed TAM	CD14, CD86, CD163, MHC II, CD64 (FcγRI)	PD-L1, TNF-α, IL-12	Exhibit either a pro-tumor M2-like phenotype or an anti-tumor M1-like phenotype. Pro tumor effects: Enhance tumor proliferation, T cell exhaustion, Immunosuppression, Decreased Immunosuppression, Suppressed T cell activation Antitumor effects: IFN-γ enhances the anti-tumor immune response by promoting the activation of M1-like macrophages, which produce pro-inflammatory cytokines such as IL-12, TNF-α. This activation can lead to increased phagocytic activity and improved tumor cell killing
Immune regulatory TAM	CD206, CD40, CD80, PD-L1, Arg1	IL-10, TGF-β, VEGF, CCL22, PDL-1	Can either promote or inhibit tumor progression Effects: Promoting Tumor Cell Survival and Proliferation, Suppressing Anti-tumor Immunity, Promotes inflammation, T cell suppression
Inflammatory cytokine-enriched TAM	CD80, CD86, CD68, MHCII	Il-1β, CXCL1/2/3/8, CCL3, CCL3L1	Pro-inflammatory activity, regulation of WNT-signaling
Lipid-associated TAM	CD36, CD163, CD206, CD80, CD40	Arg-1, XBP1, TREM2, FABP5, APOE	Promotion of Tumor Growth, Antigen processing and presentation pathways, ATP biosynthetic processes protumor effects, promotion of metastasis
Pro-angiogenic TAM	VEGF, CD206, CD163	VEGFA, PDGF, TGF-β, MMP2, MMP9, and MMP12.	Angiogenesis, Promotion of HIF pathway in tumor cell; Activate NF-kB, Notch, VEGF signaling. Protumor effects
Rresident-tissue macrophages-like TAM	CD68, CD163, Tim4, CD206	Arg1, PDL-1	Immune tolerance, Immunosuppression, promotion of tumor invasiveness
Glycolytic TAMs	CD163, CD206, Arg1	IL-1β, TNF-α, IL-6, IL-10, HIF-1α	Immunosuppression, promotion of tumor invasiveness, M2-like polarization

**Table 2:** Overlapping markers between M0 and M1- or M2-macrophages and between M2-subphenotypes.

M0***	ADGRE1	CCR2 (CD192)	CD14	CD68	CSF1R (CD115)	ITGAM (CD116)
	SPP1	PPARG	CXCR1	F4/80		
**M1**	CD11c	FCGRIA (CD64)	CD80	CD86*	iNOS*	CD40
	CXCL10	MHC-II*	TLR-2*	TLR-4*	TLR-8*	MRP8
	IL-10R*	IDA*	SPP1*	VEGF*	IL1R1*	
**M2a**	MHC-II*	SPP1*	THBS1**	IDA	CD163**	CD206**
	CD200R	CLEC7A (CD301)	CD36	CD209	FCGR3A (CD16)	MSR1 (CD204) **
	TIMD4	CD206	FIZZ1	Arg1	SPP1*	CD155
**M2b**	CD86*	MHC-II*	IL1R1*	IL-10R*	CD209*	MSR1 (CD204) **
	IDA*	PDCD1LG	THBS1			
**M2c**	TLR-2*	TLR-4*	TLR-8*	CD163**	CD206**	FCGRIA (CD64) **
	MSR1 (CD204) **	THBS1				
**M2d**	iNOS*	VEGF*	MSR1 (CD204) **			

*Overlap between M1 and M2; **Overlap between M2 subtypes; *** The overlap of between M0 and M1 or M2 phenotypes was not analysed due to the presence of transitional forms

## Abbreviations

ADAM8: A disintegrin and metalloproteinase domain 8
Angio-TAMs: pro-angiogenic tumor-associated macrophages
APCs: antigen-presenting cells
CA: colorectal adenoma
CIN: chromosomal instability
CMS: consensus molecular subtype
CRC: colorectal cancer
CSF: colony-stimulating factor
DNMT: DNA methyltransferases
EGF: epidermal growth factor
ECM: extracellular matrix
EMT: epithelial-mesenchymal transition
GM-CSF: granulocyte-macrophage colony-stimulating factor
glyc-TAMs: glycolytic tumor-associated macrophages
HDACs: histone deacetylases
HGF: hepatocyte growth factor
HIFs: hypoxia-inducible factors
HMTs: histone methyltransferases
IF: invasive front
IFN-TAMs: interferon-primed tumor-associated macrophages
IFP: interstitial fluid pressure
Inflam-TAMs: inflammatory cytokine-enriched tumor-associated macrophages
IT: intratumoral region
KCs: Kupffer cells
KLF4: Krüppel-like factor 4
LA-TAMs: lipid-associated tumor-associated macrophages
LPS: lipopolysaccharide
MAMs: Metastasis-associated macrophages
M-CSF: macrophage colony-stimulating factor
MSI: microsatellite instability
ncRNAs: non-coding RNAs
pCRC: primary CRC
PGE2: prostaglandin E2
PMN: premetastatic niche
PT: peritumor zone
Reg-TAMs: immunoregulatory tumor-associated macrophages
RLN: regional lymph node
RTM-TAMs: resident tissue macrophage-like tumor-associated macrophages
scRNAseq: single-cell RNA sequencing
SPP1: secreted phosphoprotein 1
TAMs: tumor-associated macrophages
TBM: tingible body macrophages
TGF-β: transforming growth factor-beta
TIM: tumor invasive margin
TLRs: Toll-like receptors
TME: tumor microenvironment
TF: transcription factors
VEGF: vascular endothelial growth factor

## References

[1] Yu H, Hemminki K (2020). Genetic epidemiology of colorectal cancer and associated cancers. Mutagenesis.

[2] Filip S, Vymetalkova V, Petera J (2020). Distant Metastasis in Colorectal Cancer Patients—Do We Have New Predicting Clinicopathological and Molecular Biomarkers? A Comprehensive Review. Int J Mol Sci.

[3] Guo X wen, Lei R e, Zhou Q nan, Zhang G, Hu B li, Liang Y xiao (2023). Tumor microenvironment characterization in colorectal cancer to identify prognostic and immunotherapy genes signature. BMC Cancer.

[4] Lu C, Liu Y, Ali NM, Zhang B, Cui X (2022). The role of innate immune cells in the tumor microenvironment and research progress in anti-tumor therapy. Front Immunol. 2023;13. doi:10.3389/fimmu.

[5] Wang H, Tian T, Zhang J (2021). Tumor-Associated Macrophages (TAMs) in Colorectal Cancer (CRC): From Mechanism to Therapy and Prognosis. Int J Mol Sci.

[6] Xiao L, Wang Q, Peng H (2023). Tumor-associated macrophages: new insights on their metabolic regulation and their influence in cancer immunotherapy. Front Immunol. 2023;14. doi:10.3389/fimmu.

[7] Unuvar Purcu D, Korkmaz A, Gunalp S (2022). Effect of stimulation time on the expression of human macrophage polarization markers. PLoS One.

[8] Yu Z, Zou J, Xu F (2024). Tumor-associated macrophages affect the treatment of lung cancer. Heliyon.

[9] Chanmee T, Ontong P, Konno K, Itano N (2014). Tumor-Associated Macrophages as Major Players in the Tumor Microenvironment. Cancers (Basel).

[10] Hourani T, Holden JA, Li W, Lenzo JC, Hadjigol S, O'Brien-Simpson NM (2021). Tumor Associated Macrophages: Origin, Recruitment, Phenotypic Diversity, and Targeting. Front Oncol. 2021;11. doi:10.3389/fonc.

[11] Boutilier AJ, Elsawa SF (2021). Macrophage Polarization States in the Tumor Microenvironment. Int J Mol Sci.

[12] Bai R, Li Y, Jian L, Yang Y, Zhao L, Wei M (2022). The hypoxia-driven crosstalk between tumor and tumor-associated macrophages: mechanisms and clinical treatment strategies. Mol Cancer.

[13] Vitale I, Manic G, Coussens LM, Kroemer G, Galluzzi L (2019). Macrophages and Metabolism in the Tumor Microenvironment. Cell Metab.

[14] Kerneur C, Cano CE, Olive D (2022). Major pathways involved in macrophage polarization in cancer. Front Immunol. 2022;13. doi:10.3389/fimmu.

[15] Yang Q, Guo N, Zhou Y, Chen J, Wei Q, Han M (2020). The role of tumor-associated macrophages (TAMs) in tumor progression and relevant advance in targeted therapy. Acta Pharm Sin B.

[16] Cheruku S, Rao V, Pandey R, Rao Chamallamudi M, Velayutham R, Kumar N (2023). Tumor-associated macrophages employ immunoediting mechanisms in colorectal tumor progression: Current research in Macrophage repolarization immunotherapy. Int Immunopharmacol.

[17] Cao M, Wang Z, Lan W (2024). The roles of tissue resident macrophages in health and cancer. Exp Hematol Oncol.

[18] Wang H, Yung MMH, Ngan HYS, Chan KKL, Chan DW (2021). The Impact of the Tumor Microenvironment on Macrophage Polarization in Cancer Metastatic Progression. Int J Mol Sci.

[19] Waldner MJ, Neurath MF (2023). TGFβ and the Tumor Microenvironment in Colorectal Cancer. Cells.

[20] Afik R, Zigmond E, Vugman M (2016). Tumor macrophages are pivotal constructors of tumor collagenous matrix. Journal of Experimental Medicine.

[21] De Martino D, Bravo-Cordero JJ (2023). Collagens in Cancer: Structural Regulators and Guardians of Cancer Progression. Cancer Res.

[22] Kainulainen K, Takabe P, Heikkinen S (2022). M1 Macrophages Induce Protumor Inflammation in Melanoma Cells through TNFR-NF-κB Signaling. Journal of Investigative Dermatology.

[23] Gao J, Liang Y, Wang L (2022). Shaping Polarization Of Tumor-Associated Macrophages In Cancer Immunotherapy. Front Immunol. 2022;13. doi:10.3389/fimmu.

[24] Wang S, Wang J, Chen Z (2024). Targeting M2-like tumor-associated macrophages is a potential therapeutic approach to overcome antitumor drug resistance. NPJ Precis Oncol.

[25] Rőszer T (2015). Understanding the Mysterious M2 Macrophage through Activation Markers and Effector Mechanisms. Mediators Inflamm.

[26] Li P, Ma C, Li J (2022). Proteomic characterization of four subtypes of M2 macrophages derived from human THP-1 cells. Journal of Zhejiang University-SCIENCE B.

[27] Yuan A, Hsiao YJ, Chen HY (2015). Opposite Effects of M1 and M2 Macrophage Subtypes on Lung Cancer Progression. Sci Rep.

[28] Fridman WH, Pagès F, Sautès-Fridman C, Galon J (2012). The immune contexture in human tumours: impact on clinical outcome. Nat Rev Cancer.

[29] Graff JW, Dickson AM, Clay G, McCaffrey AP, Wilson ME (2012). Identifying Functional MicroRNAs in Macrophages with Polarized Phenotypes. Journal of Biological Chemistry.

[30] Mendoza-Coronel E, Ortega E (2017). Macrophage Polarization Modulates FcγR- and CD13-Mediated Phagocytosis and Reactive Oxygen Species Production, Independently of Receptor Membrane Expression. Front Immunol. 2017;8. doi:10.3389/fimmu.

[31] Fuchs AL, Costello SM, Schiller SM, Tripet BP, Copié V (2024). Primary Human M2 Macrophage Subtypes Are Distinguishable by Aqueous Metabolite Profiles. Int J Mol Sci.

[32] Italiani P, Boraschi D (2014). From Monocytes to M1/M2 Macrophages: Phenotypical vs. Functional Differentiation. *Front Immunol*. 2014;5. doi:10.3389/fimmu.

[33] Zhang W, Wang M, Ji C, Liu X, Gu B, Dong T (2024). Macrophage polarization in the tumor microenvironment: Emerging roles and therapeutic potentials. Biomedicine & Pharmacotherapy.

[34] ChÃvez-GalÃn L, Olleros ML, Vesin D, Garcia I (2015). Much More than M1 and M2 Macrophages, There are also CD169+ and TCR+ Macrophages. Front Immunol. 2015;6. doi:10.3389/fimmu.

[35] Edin S, Wikberg ML, Dahlin AM (2012). The Distribution of Macrophages with a M1 or M2 Phenotype in Relation to Prognosis and the Molecular Characteristics of Colorectal Cancer. PLoS One.

[36] Liu J, Geng X, Hou J, Wu G (2021). New insights into M1/M2 macrophages: key modulators in cancer progression. Cancer Cell Int.

[37] Hoffman D, Tevet Y, Trzebanski S (2021). A non-classical monocyte-derived macrophage subset provides a splenic replication niche for intracellular Salmonella. Immunity.

[38] Li C, Luo X, Lin Y (2015). A Higher Frequency of CD14+CD169+ Monocytes/Macrophages in Patients with Colorectal Cancer. PLoS One.

[39] Saito Y, Fujiwara Y, Miyamoto Y (2023). <scp>CD169</scp> ^+^ sinus macrophages in regional lymph nodes do not predict mismatch-repair status of patients with colorectal cancer. Cancer Med.

[40] Koelzer VH, Canonica K, Dawson H (2015). Phenotyping of tumor-associated macrophages in colorectal cancer: Impact on single cell invasion (tumor budding) and clinicopathological outcome. Oncoimmunology. 2016;5(4). doi:10.1080/2162402X.

[41] Strizova Z, Benesova I, Bartolini R (2023). M1/M2 macrophages and their overlaps - myth or reality?. Clin Sci.

[42] Ma RY, Black A, Qian BZ (2022). Macrophage diversity in cancer revisited in the era of single-cell omics. Trends Immunol.

[43] Nasir I, McGuinness C, Poh AR, Ernst M, Darcy PK, Britt KL (2023). Tumor macrophage functional heterogeneity can inform the development of novel cancer therapies. Trends Immunol.

[44] Yi C, Li Z, Zhao Q (2024). Single-Cell RNA Sequencing Pro-angiogenic Macrophage Profiles Reveal Novel Prognostic Biomarkers and Therapeutic Targets for Osteosarcoma. Biochem Genet.

[45] Wei C, Ma Y, Wang M (2024). Tumor-associated macrophage clusters linked to immunotherapy in a pan-cancer census. NPJ Precis Oncol.

[46] Bain CC, Bravo-Blas A, Scott CL (2014). Constant replenishment from circulating monocytes maintains the macrophage pool in the intestine of adult mice. Nat Immunol.

[47] Smith PD, Smythies LE, Shen R, Greenwell-Wild T, Gliozzi M, Wahl SM (2011). Intestinal macrophages and response to microbial encroachment. Mucosal Immunol.

[48] Bain CC, Scott CL, Uronen-Hansson H (2013). Resident and pro-inflammatory macrophages in the colon represent alternative context-dependent fates of the same Ly6Chi monocyte precursors. Mucosal Immunol.

[49] Hadis U, Wahl B, Schulz O (2011). Intestinal Tolerance Requires Gut Homing and Expansion of FoxP3+ Regulatory T Cells in the Lamina Propria. Immunity.

[50] Zigmond E, Bernshtein B, Friedlander G (2014). Macrophage-Restricted Interleukin-10 Receptor Deficiency, but Not IL-10 Deficiency, Causes Severe Spontaneous Colitis. Immunity.

[51] Kim YG, Udayanga KGS, Totsuka N, Weinberg JB, Núñez G, Shibuya A (2014). Gut Dysbiosis Promotes M2 Macrophage Polarization and Allergic Airway Inflammation via Fungi-Induced PGE2. Cell Host Microbe.

[52] Guillaume J, Leufgen A, Hager FT, Pabst O, Cerovic V (2023). MHCII expression on gut macrophages supports T cell homeostasis and is regulated by microbiota and ontogeny. Sci Rep.

[53] Medina-Contreras O, Geem D, Laur O (2011). CX3CR1 regulates intestinal macrophage homeostasis, bacterial translocation, and colitogenic Th17 responses in mice. Journal of Clinical Investigation.

[54] Mori K, Haraguchi S, Hiori M, Shimada J, Ohmori Y (2015). Tumor-associated macrophages in oral premalignant lesions coexpress CD163 and STAT1 in a Th1-dominated microenvironment. BMC Cancer.

[55] Selvakumar B, Sekar P, Samsudin AR (2024). Intestinal macrophages in pathogenesis and treatment of gut leakage: current strategies and future perspectives. J Leukoc Biol.

[56] Bernardo D, Marin AC, Fernández-Tomé S (2018). Human intestinal pro-inflammatory CD11chighCCR2+CX3CR1+ macrophages, but not their tolerogenic CD11c-CCR2-CX3CR1- counterparts, are expanded in inflammatory bowel disease. Mucosal Immunol.

[57] Bain CC, Schridde A (2018). Origin, Differentiation, and Function of Intestinal Macrophages. Front Immunol. 2018;9. doi:10.3389/fimmu.

[58] Bujko A, Atlasy N, Landsverk OJB (2018). Transcriptional and functional profiling defines human small intestinal macrophage subsets. Journal of Experimental Medicine.

[59] Gurwicz N, Stoler-Barak L, Schwan N, Bandyopadhyay A, Meyer-Hermann M, Shulman Z (2023). Tingible body macrophages arise from lymph node-resident precursors and uptake B cells by dendrites. Journal of Experimental Medicine.

[60] Smith TD, Tse MJ, Read EL, Liu WF (2016). Regulation of macrophage polarization and plasticity by complex activation signals. Integrative Biology.

[61] Smith JP, Burton GF, Tew JG, Szakal AK (1998). Tinigible Body Macrophages in Regulation of Germinal Center Reactions. J Immunol Res.

[62] Imtiyaz HZ, Williams EP, Hickey MM (2010). Hypoxia-inducible factor 2α regulates macrophage function in mouse models of acute and tumor inflammation. Journal of Clinical Investigation.

[63] Zhang H, Wang X, Zhang J (2023). Crosstalk between gut microbiota and gut resident macrophages in inflammatory bowel disease. J Transl Int Med.

[64] Grivennikov SI, Wang K, Mucida D (2012). Adenoma-linked barrier defects and microbial products drive IL-23/IL-17-mediated tumour growth. Nature.

[65] Wierzbicki J, Bednarz-Misa I, Lewandowski Ł (2024). Macrophage Inflammatory Proteins (MIPs) Contribute to Malignant Potential of Colorectal Polyps and Modulate Likelihood of Cancerization Associated with Standard Risk Factors. Int J Mol Sci.

[66] Li W, Chen F, Gao H (2023). Cytokine concentration in peripheral blood of patients with colorectal cancer. Front Immunol. 2023;14. doi:10.3389/fimmu.

[67] Lepsenyi M, Algethami N, Al-Haidari AA (2021). CXCL2-CXCR2 axis mediates αV integrin-dependent peritoneal metastasis of colon cancer cells. Clin Exp Metastasis.

[68] Zhang Q, Sioud M (2023). Tumor-Associated Macrophage Subsets: Shaping Polarization and Targeting. Int J Mol Sci.

[69] Wu TH, Li YY, Wu TL (2014). Culture supernatants of different colon cancer cell lines induce specific phenotype switching and functional alteration of THP-1 cells. Cell Immunol.

[70] Lundholm M, Hägglöf C, Wikberg ML (2015). Secreted Factors from Colorectal and Prostate Cancer Cells Skew the Immune Response in Opposite Directions. Sci Rep.

[71] Chai N, Xiong Y, Zhang Y (2021). YYFZBJS inhibits colorectal tumorigenesis by remodeling gut microbiota and influence on M2 macrophage polarization *in vivo* and *in vitro*. Am J Cancer Res.

[72] Soncin I, Sheng J, Chen Q (2018). The tumour microenvironment creates a niche for the self-renewal of tumour-promoting macrophages in colon adenoma. Nat Commun.

[73] Wang B, Li Q, Qin L, Zhao S, Wang J, Chen X (2011). Transition of tumor-associated macrophages from MHC class IIhi to MHC class IIlow mediates tumor progression in mice. BMC Immunol.

[74] TANIYAMA D, TANIYAMA K, KURAOKA K (2019). CD204-Positive Tumor-associated Macrophages Relate to Malignant Transformation of Colorectal Adenoma. Anticancer Res.

[75] Zhu M, Bai L, Liu X (2022). Silence of a dependence receptor CSF1R in colorectal cancer cells activates tumor-associated macrophages. J Immunother Cancer.

[76] Siskova A, Cervena K, Kral J, Hucl T, Vodicka P, Vymetalkova V (2020). Colorectal Adenomas—Genetics and Searching for New Molecular Screening Biomarkers. Int J Mol Sci.

[77] Hayes BH, Wang M, Zhu H (2023). Chromosomal instability can favor macrophage-mediated immune response and induce a broad, vaccination-like anti-tumor IgG response. Preprint posted online April 4.

[78] Niu Y, Chen J, Qiao Y (2022). Epigenetic Modifications in Tumor-Associated Macrophages: A New Perspective for an Old Foe. Front Immunol. 2022;13. doi:10.3389/fimmu.

[79] Tang RZ, Zhu JJ, Yang FF (2019). DNA methyltransferase 1 and Krüppel-like factor 4 axis regulates macrophage inflammation and atherosclerosis. J Mol Cell Cardiol.

[80] Yang X, Wang X, Liu D, Yu L, Xue B, Shi H (2014). Epigenetic Regulation of Macrophage Polarization by DNA Methyltransferase 3b. Molecular Endocrinology.

[81] Zhang Y, Liu H (2025). Aberrant DNMT1-mediated DACH1 methylation is associated with colorectal adenoma-to-carcinoma progression. Exp Biol Med. 2025;250. doi:10.3389/ebm.

[82] Ahadi A (2020). The significance of microRNA deregulation in colorectal cancer development and the clinical uses as a diagnostic and prognostic biomarker and therapeutic agent. Noncoding RNA Res.

[83] Ling Q, Fang J, Zhai C (2023). Berberine induces SOCS1 pathway to reprogram the M1 polarization of macrophages via miR-155-5p in colitis-associated colorectal cancer. Eur J Pharmacol.

[84] Shinohara H, Kuranaga Y, Kumazaki M (2017). Regulated Polarization of Tumor-Associated Macrophages by miR-145 via Colorectal Cancer-Derived Extracellular Vesicles. The Journal of Immunology.

[85] Crame EE, Nourmohammadi S, Wardill HR, Coller JK, Bowen JM (2023). Contribution of TLR4 to colorectal tumor microenvironment, etiology and prognosis. J Cancer Res Clin Oncol.

[86] Yang M, Liu J, Piao C, Shao J, Du J (2015). ICAM-1 suppresses tumor metastasis by inhibiting macrophage M2 polarization through blockade of efferocytosis. Cell Death Dis.

[87] Arai H, Gandhi N, Battaglin F (2024). Role of *CD47* gene expression in colorectal cancer: a comprehensive molecular profiling study. J Immunother Cancer.

[88] Deng H, Wang G, Zhao S (2023). New hope for tumor immunotherapy: the macrophage-related “do not eat me” signaling pathway. Front Pharmacol. 2023;14. doi:10.3389/fphar.

[89] Zhang Y, Sime W, Juhas M, Sjölander A (2013). Crosstalk between colon cancer cells and macrophages via inflammatory mediators and CD47 promotes tumour cell migration. Eur J Cancer.

[90] Montalbán-Hernández K, Cantero-Cid R, Casalvilla-Dueñas JC (2022). Colorectal Cancer Stem Cells Fuse with Monocytes to Form Tumour Hybrid Cells with the Ability to Migrate and Evade the Immune System. Cancers (Basel).

[91] Stopforth RJ, Ward ES (2020). The Role of Antigen Presentation in Tumor-Associated Macrophages. Crit Rev Immunol.

[92] Li C, Song J, Guo Z (2022). EZH2 Inhibitors Suppress Colorectal Cancer by Regulating Macrophage Polarization in the Tumor Microenvironment. Front Immunol. 2022;13. doi:10.3389/fimmu.

[93] Velázquez KT, Enos RT, McClellan JL (2016). MicroRNA-155 deletion promotes tumorigenesis in the azoxymethane-dextran sulfate sodium model of colon cancer. American Journal of Physiology-Gastrointestinal and Liver Physiology.

[94] Hou S, Zhao Y, Chen J, Lin Y, Qi X (2024). Tumor-associated macrophages in colorectal cancer metastasis: molecular insights and translational perspectives. J Transl Med.

[95] Mantovani A, Barajon I, Garlanda C (2018). <scp>IL</scp> -1 and <scp>IL</scp> -1 regulatory pathways in cancer progression and therapy. Immunol Rev.

[96] Wu Y, Zhang S, Yan J (2020). IRF1 association with tumor immune microenvironment and use as a diagnostic biomarker for colorectal cancer recurrence. *Oncol Lett*. Published online January 10, 2020. doi:10.3892/ol.

[97] XIE C, LIU C, WU B (2016). Effects of IRF1 and IFN-β interaction on the M1 polarization of macrophages and its antitumor function. Int J Mol Med.

[98] Bhat AA, Nisar S, Singh M (2022). Cytokine- and chemokine-induced inflammatory colorectal tumor microenvironment: Emerging avenue for targeted therapy. Cancer Commun.

[99] Akter R, Park R, Lee SK (2024). Upregulation of EMR1 (ADGRE1) by Tumor-Associated Macrophages Promotes Colon Cancer Progression by Activating the JAK2/STAT1,3 Signaling Pathway in Tumor Cells. Int J Mol Sci.

[100] Dorrington MG, Fraser IDC (2019). NF-κB Signaling in Macrophages: Dynamics, Crosstalk, and Signal Integration. Front Immunol. 2019;10. doi:10.3389/fimmu.

[101] Chistiakov DA, Myasoedova VA, Revin V V, Orekhov AN, Bobryshev Y V (2018). The impact of interferon-regulatory factors to macrophage differentiation and polarization into M1 and M2. Immunobiology.

[102] Huang L, Zhao Y, Shan M (2023). Targeting crosstalk of STAT3 between tumor-associated M2 macrophages and Tregs in colorectal cancer. Cancer Biol Ther. 2023;24(1). doi:10.1080/15384047.

[103] Cao H, Zhang J, Liu H (2016). IL-13/STAT6 signaling plays a critical role in the epithelial-mesenchymal transition of colorectal cancer cells. Oncotarget.

[104] Cheng C, Huang C, Ma TT (2014). SOCS1 hypermethylation mediated by DNMT1 is associated with lipopolysaccharide-induced inflammatory cytokines in macrophages. Toxicol Lett.

[105] Shao X, Xu P, Ji L (2023). Low-dose decitabine promotes M2 macrophage polarization in patients with primary immune thrombocytopenia via enhancing KLF4 binding to PPARγ promoter. Clin Transl Med.

[106] Tan L, Shi YG (2012). Tet family proteins and 5-hydroxymethylcytosine in development and disease. Development.

[107] He S, Song W, Cui S (2023). Modulation of miR-146b by N6-methyladenosine modification remodels tumor-associated macrophages and enhances anti-PD-1 therapy in colorectal cancer. Cellular Oncology.

[108] Chen C, Liu T, Tang Y, Luo G, Liang G, He W (2023). Epigenetic regulation of macrophage polarization in wound healing. Burns Trauma.

[109] Vadevoo SMP, Gunassekaran GR, Yoo J Do (2022). Epigenetic therapy reprograms M2-type tumor-associated macrophages into an M1-like phenotype by upregulating miR-7083-5p. Front Immunol. 2022;13. doi:10.3389/fimmu.

[110] Lu Y, Ma S, Chan YT, Wu Y, Feng Y, Wang N (2025). Epigenetic orchestration of cancer-immune dynamics: mechanisms, technologies, and clinical advancements. *J Adv Res*. Published online September.

[111] Zhu W, Liu L, Wu J (2024). SMYD3 activates the TCA cycle to promote M1-M2 conversion in macrophages. Int Immunopharmacol.

[112] Strachowska M, Robaszkiewicz A (2024). Characteristics of anticancer activity of CBP/p300 inhibitors - Features of their classes, intracellular targets and future perspectives of their application in cancer treatment. Pharmacol Ther.

[113] Zhang W, Ge L, Zhang Y (2025). Targeted intervention of tumor microenvironment with HDAC inhibitors and their combination therapy strategies. Eur J Med Res.

[114] Yi B, Dai K, Yan Z, Yin Z (2022). Circular RNA PLCE1 promotes epithelial mesenchymal transformation, glycolysis in colorectal cancer and M2 polarization of tumor-associated macrophages. Bioengineered.

[115] Liu J, Chen Z, Xiang J, Gu X (2018). MicroRNA-155 acts as a tumor suppressor in colorectal cancer by targeting CTHRC1 inïvitro. *Oncol Lett*. Published online February 16, 2018. doi:10.3892/ol.

[116] Louafi F, Martinez-Nunez RT, Sanchez-Elsner T (2010). MicroRNA-155 Targets SMAD2 and Modulates the Response of Macrophages to Transforming Growth Factor-β. Journal of Biological Chemistry.

[117] Sun L, Chen B, Wu J (2020). Epigenetic Regulation of a Disintegrin and Metalloproteinase (ADAM) Transcription in Colorectal Cancer Cells: Involvement of β-Catenin, BRG1, and KDM4. Front Cell Dev Biol. 2020;8. doi:10.3389/fcell.

[118] Mierke CT (2023). The versatile roles of ADAM8 in cancer cell migration, mechanics, and extracellular matrix remodeling. Front Cell Dev Biol. 2023;11. doi:10.3389/fcell.

[119] Li Y, Chen Z, Han J, Ma X, Zheng X, Chen J (2022). Functional and Therapeutic Significance of Tumor-Associated Macrophages in Colorectal Cancer. Front Oncol. 2022;12. doi:10.3389/fonc.

[120] Jahandideh A, Yarizadeh M, Noei-Khesht Masjedi M (2023). Macrophage's role in solid tumors: two edges of a sword. Cancer Cell Int.

[121] Zhong X, Chen B, Yang Z (2018). The Role of Tumor-Associated Macrophages in Colorectal Carcinoma Progression. Cellular Physiology and Biochemistry.

[122] Cortese N, Soldani C, Franceschini B (2019). Macrophages in Colorectal Cancer Liver Metastases. Cancers (Basel).

[123] Scheurlen KM, Billeter AT, O'Brien SJ, Galandiuk S (2020). Metabolic dysfunction and early-onset colorectal cancer - how macrophages build the bridge. Cancer Med.

[124] Li J, Li L, Li Y (2020). Tumor-associated macrophage infiltration and prognosis in colorectal cancer: systematic review and meta-analysis. Int J Colorectal Dis.

[125] Kim HD, Kim SY, Kim J (2022). Dynamic increase of M2 macrophages is associated with disease progression of colorectal cancers following cetuximab-based treatment. Sci Rep.

[126] Konstantinov AS, Kovaleva O V, Samoilova D V, Shelekhova K V (2022). Role of macrophages in progression of colorectal cancer: a contrast with the traditional paradigm. Int J Clin Exp Pathol.

[127] Xu G, Mo Y, Li J, Wei Q, Zhou F, Chen J (2023). Two tripartite classification systems of CD86+ and CD206+ macrophages are significantly associated with tumor recurrence in stage II-III colorectal cancer. Front Immunol. 2023;14. doi:10.3389/fimmu.

[128] Wang X, Yuwen T jiao, Zhong Y, Li ZG, Wang XY (2023). A new method for predicting the prognosis of colorectal cancer patients through a combination of multiple tumor-associated macrophage markers at the invasive front. Heliyon.

[129] Modak M, Mattes AK, Reiss D (2022). CD206+ tumor-associated macrophages cross-present tumor antigen and drive antitumor immunity. JCI Insight.

[130] Magnussen AL, Mills IG (2021). Vascular normalisation as the stepping stone into tumour microenvironment transformation. Br J Cancer.

[131] Qi J, Sun H, Zhang Y (2022). Single-cell and spatial analysis reveal interaction of FAP+ fibroblasts and SPP1+ macrophages in colorectal cancer. Nat Commun.

[132] Ozato Y, Kojima Y, Kobayashi Y (2023). Spatial and single-cell transcriptomics decipher the cellular environment containing HLA-G+ cancer cells and SPP1+ macrophages in colorectal cancer. Cell Rep.

[133] Lin D, Zheng T, Huang S, Liu R, Guan S, Zhang Z (2024). Identification of a novel macrophage-related prognostic signature in colorectal cancer. Sci Rep.

[134] Li S, Xu F, Zhang J (2017). Tumor-associated macrophages remodeling EMT and predicting survival in colorectal carcinoma. Oncoimmunology. 2018;7(2). doi:10.1080/2162402X.

[135] Pinto ML, Rios E, Durães C (2019). The Two Faces of Tumor-Associated Macrophages and Their Clinical Significance in Colorectal Cancer. Front Immunol. 2019;10. doi:10.3389/fimmu.

[136] Tang J, Ming L, Qin F (2024). The heterogeneity of tumour-associated macrophages contributes to the clinical outcomes and indications for immune checkpoint blockade in colorectal cancer patients. Immunobiology.

[137] Yang C, Wei C, Wang S (2019). Elevated CD163 ^+^ /CD68 ^+^ Ratio at Tumor Invasive Front is Closely Associated with Aggressive Phenotype and Poor Prognosis in Colorectal Cancer. Int J Biol Sci.

[138] Xue T, Yan K, Cai Y (2021). Prognostic significance of CD163+ tumor-associated macrophages in colorectal cancer. World J Surg Oncol.

[139] Schnell A, Schmidl C, Herr W, Siska PJ (2018). The Peripheral and Intratumoral Immune Cell Landscape in Cancer Patients: A Proxy for Tumor Biology and a Tool for Outcome Prediction. Biomedicines.

[140] Mezheyeuski A, Micke P, Martín-Bernabé A (2021). The Immune Landscape of Colorectal Cancer. Cancers (Basel).

[141] Inagaki K, Kunisho S, Takigawa H (2021). Role of tumor-associated macrophages at the invasive front in human colorectal cancer progression. Cancer Sci.

[142] Bund T, Nikitina E, Chakraborty D (2021). Analysis of chronic inflammatory lesions of the colon for BMMF Rep antigen expression and CD68 macrophage interactions. Proceedings of the National Academy of Sciences.

[143] Ye J, Guo W, Wang C (2023). Peritumoral Immune-suppressive Mechanisms Impede Intratumoral Lymphocyte Infiltration into Colorectal Cancer Liver versus Lung Metastases. Cancer Research Communications.

[144] Cau F, Gerosa C, Ziranu P (2023). L1CAM EXPRESSION IN THE PERITUMORAL STROMAL CELLS OF THE MICROENVIRONMENT OF COLORECTAL CANCER: A NEW TARGET FOR ONCOLOGISTS?. Annals of Research in Oncology.

[145] Soldevilla B, Carretero-Puche C, Gomez-Lopez G (2019). The correlation between immune subtypes and consensus molecular subtypes in colorectal cancer identifies novel tumour microenvironment profiles, with prognostic and therapeutic implications. Eur J Cancer.

[146] ten Hoorn S, de Back TR, Sommeijer DW, Vermeulen L (2022). Clinical Value of Consensus Molecular Subtypes in Colorectal Cancer: A Systematic Review and Meta-Analysis. JNCI: Journal of the National Cancer Institute.

[147] Guinney J, Dienstmann R, Wang X (2015). The consensus molecular subtypes of colorectal cancer. Nat Med.

[148] Picard E, Verschoor CP, Ma GW, Pawelec G (2020). Relationships Between Immune Landscapes, Genetic Subtypes and Responses to Immunotherapy in Colorectal Cancer. Front Immunol. 2020;11. doi:10.3389/fimmu.

[149] Karaman S, Detmar M (2014). Mechanisms of lymphatic metastasis. Journal of Clinical Investigation.

[150] Jones D, Pereira ER, Padera TP (2018). Growth and Immune Evasion of Lymph Node Metastasis. Front Oncol. 2018;8. doi:10.3389/fonc.

[151] Gharavi AT, Hanjani NA, Movahed E, Doroudian M (2022). The role of macrophage subtypes and exosomes in immunomodulation. Cell Mol Biol Lett.

[152] Wang Y, Wang J, Yang C (2021). A study of the correlation between M2 macrophages and lymph node metastasis of colorectal carcinoma. World J Surg Oncol.

[153] Ji H, Hu C, Yang X (2023). Lymph node metastasis in cancer progression: molecular mechanisms, clinical significance and therapeutic interventions. Signal Transduct Target Ther.

[154] Hompland T, Ellingsen C, Øvrebø KM, Rofstad EK (2012). Interstitial Fluid Pressure and Associated Lymph Node Metastasis Revealed in Tumors by Dynamic Contrast-Enhanced MRI. Cancer Res.

[155] Li R, Serrano JC, Xing H (2018). Interstitial flow promotes macrophage polarization toward an M2 phenotype. Mol Biol Cell.

[156] Viola MF, Boeckxstaens G (2020). Intestinal resident macrophages: Multitaskers of the gut. Neurogastroenterology & Motility.

[157] Cao W, Peters JH, Nieman D, Sharma M, Watson T, Yu J (2015). Macrophage subtype predicts lymph node metastasis in oesophageal adenocarcinoma and promotes cancer cell invasion *in vitro*. Br J Cancer.

[158] Fan L, Ma LX, Zhou P, Shao ZM (2022). Atlas of immune cell infiltration in breast cancer—high M2 macrophage and low native B cellproportions are associated with poor survival. Annals of Breast Surgery.

[159] Illemann M, Bird N, Majeed A (2006). MMP-9 Is Differentially Expressed in Primary Human Colorectal Adenocarcinomas and Their Metastases. Molecular Cancer Research.

[160] Xu J, Gao Y, Ding Y (2024). Correlation between Tregs and ICOS-induced M2 macrophages polarization in colorectal cancer progression. Front Oncol. 2024;14. doi:10.3389/fonc.

[161] Komohara Y, Ohnishi K, Takeya M (2017). Possible functions of <scp>CD</scp> 169-positive sinus macrophages in lymph nodes in anti-tumor immune responses. Cancer Sci.

[162] Emile MH, Emile SH, El-Karef AA, Ebrahim MA, Mohammed IE, Ibrahim DA (2024). Association between the expression of epithelial-mesenchymal transition (EMT)-related markers and oncologic outcomes of colorectal cancer. Updates Surg.

[163] Bhome R, Emaduddin M, James V (2022). Epithelial to mesenchymal transition influences fibroblast phenotype in colorectal cancer by altering miR-200 levels in extracellular vesicles. J Extracell Vesicles.

[164] Mittal V (2018). Epithelial Mesenchymal Transition in Tumor Metastasis. Annual Review of Pathology: Mechanisms of Disease.

[165] Cai J, Xia L, Li J, Ni S, Song H, Wu X (2019). Tumor-Associated Macrophages Derived TGF-β-Induced Epithelial to Mesenchymal Transition in Colorectal Cancer Cells through Smad2,3-4/Snail Signaling Pathway. Cancer Res Treat.

[166] Gazzillo A, Polidoro MA, Soldani C, Franceschini B, Lleo A, Donadon M (2022). Relationship between Epithelial-to-Mesenchymal Transition and Tumor-Associated Macrophages in Colorectal Liver Metastases. Int J Mol Sci.

[167] Li Y, Zhu G, Zhai H (2018). Simultaneous stimulation with tumor necrosis factor-α and transforming growth factor-β1 induces epithelial-mesenchymal transition in colon cancer cells via the NF-κB pathway. *Oncol Lett*. Published online March 12, 2018. doi:10.3892/ol.

[168] Zhang Y, Zhao Y, Li Q, Wang Y (2021). Macrophages, as a Promising Strategy to Targeted Treatment for Colorectal Cancer Metastasis in Tumor Immune Microenvironment. Front Immunol. 2021;12. doi:10.3389/fimmu.

[169] Jager NA, Wallis de Vries BM, Hillebrands JL (2016). Distribution of Matrix Metalloproteinases in Human Atherosclerotic Carotid Plaques and Their Production by Smooth Muscle Cells and Macrophage Subsets. Mol Imaging Biol.

[170] Mantovani A, Allavena P, Marchesi F, Garlanda C (2022). Macrophages as tools and targets in cancer therapy. Nat Rev Drug Discov.

[171] Zhou Q, Peng RQ, Wu XJ (2010). The density of macrophages in the invasive front is inversely correlated to liver metastasis in colon cancer. J Transl Med.

[172] Zajac E, Schweighofer B, Kupriyanova TA (2013). Angiogenic capacity of M1- and M2-polarized macrophages is determined by the levels of TIMP-1 complexed with their secreted proMMP-9. Blood.

[173] Bi Y, Shirure VS, Liu R (2020). Tumor-on-a-chip platform to interrogate the role of macrophages in tumor progression. Integrative Biology.

[174] Feng Y, Qiao S, Chen J (2024). M2-Type Macrophages and Cancer-Associated Fibroblasts Combine to Promote Colorectal Cancer Liver Metastases. Onco Targets Ther.

[175] Winkler J, Abisoye-Ogunniyan A, Metcalf KJ, Werb Z (2020). Concepts of extracellular matrix remodelling in tumour progression and metastasis. Nat Commun.

[176] Ma S, Zhao Y, Liu X (2022). CD163 as a Potential Biomarker in Colorectal Cancer for Tumor Microenvironment and Cancer Prognosis: A Swedish Study from Tissue Microarrays to Big Data Analyses. Cancers (Basel).

[177] Arwert EN, Harney AS, Entenberg D (2018). A Unidirectional Transition from Migratory to Perivascular Macrophage Is Required for Tumor Cell Intravasation. Cell Rep.

[178] Garg P, Jallepalli VR, Verma S (2024). Unravelling the CXCL12/CXCR4 Axis in breast cancer: Insights into metastasis, microenvironment interactions, and therapeutic opportunities. Human Gene.

[179] Aras S, Zaidi MR (2017). TAMeless traitors: macrophages in cancer progression and metastasis. Br J Cancer.

[180] Wang D, Sun H, Wei J, Cen B, DuBois RN (2017). CXCL1 Is Critical for Premetastatic Niche Formation and Metastasis in Colorectal Cancer. Cancer Res.

[181] Zhong Q, Fang Y, Lai Q (2020). CPEB3 inhibits epithelial-mesenchymal transition by disrupting the crosstalk between colorectal cancer cells and tumor-associated macrophages via IL-6R/STAT3 signaling. Journal of Experimental & Clinical Cancer Research.

[182] Spina A, De Pasquale V, Cerulo G (2015). HGF/c-MET Axis in Tumor Microenvironment and Metastasis Formation. Biomedicines.

[183] Zhang Y, Xia M, Jin K (2018). Function of the c-Met receptor tyrosine kinase in carcinogenesis and associated therapeutic opportunities. Mol Cancer.

[184] Shao Y, Chen T, Zheng X (2018). Colorectal cancer-derived small extracellular vesicles establish an inflammatory premetastatic niche in liver metastasis. Carcinogenesis.

[185] Hoshino A, Costa-Silva B, Shen TL (2015). Tumour exosome integrins determine organotropic metastasis. Nature.

[186] Niu Y, Yang W, Qian H, Sun Y (2022). Intracellular and extracellular factors of colorectal cancer liver metastasis: a pivotal perplex to be fully elucidated. Cancer Cell Int.

[187] Wu Y, Yang S, Ma J (2022). Spatiotemporal Immune Landscape of Colorectal Cancer Liver Metastasis at Single-Cell Level. Cancer Discov.

[188] Wang Y, Jia J, Wang F (2024). Pre-metastatic niche: formation, characteristics and therapeutic implication. Signal Transduct Target Ther.

[189] Zhao S, Mi Y, Guan B (2020). Tumor-derived exosomal miR-934 induces macrophage M2 polarization to promote liver metastasis of colorectal cancer. J Hematol Oncol.

[190] Kolios G (2006). Role of Kupffer cells in the pathogenesis of liver disease. World J Gastroenterol.

[191] García-Pérez R, Ferrer Fábrega J, Varona-Bosque A (2018). Role of Kupffer cells in the progression of CRC liver metastases after the first stage of ALPPS. Sci Rep.

[192] Khanduri I, Maru DM, Parra ER (2023). Exploratory study of macrophage polarization and spatial distribution in colorectal cancer liver metastasis: a pilot study. Front Immunol. 2023;14. doi:10.3389/fimmu.

[193] Li S, Hao L, Hu X (2024). Biological Roles and Clinical Therapeutic Applications of Tumor-Associated Macrophages in Colorectal Liver Metastasis. J Inflamm Res.

[194] Trovato R, Canè S, Petrova V, Sartoris S, Ugel S, De Sanctis F (2020). The Engagement Between MDSCs and Metastases: Partners in Crime. Front Oncol. 2020;10. doi:10.3389/fonc.

[195] Clawson GA, Matters GL, Xin P (2015). Macrophage-Tumor Cell Fusions from Peripheral Blood of Melanoma Patients. PLoS One.

[196] Kitamura T, Qian BZ, Soong D (2015). CCL2-induced chemokine cascade promotes breast cancer metastasis by enhancing retention of metastasis-associated macrophages. Journal of Experimental Medicine.

[197] Tu W, Gong J, Zhou Z, Tian D, Wang Z (2021). TCF4 enhances hepatic metastasis of colorectal cancer by regulating tumor-associated macrophage via CCL2/CCR2 signaling. Cell Death Dis.

[198] Grossman JG, Nywening TM, Belt BA (2018). Recruitment of CCR2 ^+^ tumor associated macrophage to sites of liver metastasis confers a poor prognosis in human colorectal cancer. Oncoimmunology.

[199] Ou B, Cheng X, Xu Z (2019). A positive feedback loop of β-catenin/CCR2 axis promotes regorafenib resistance in colorectal cancer. Cell Death Dis.

[200] Zhang R, Qi F, Zhao F (2019). Cancer-associated fibroblasts enhance tumor-associated macrophages enrichment and suppress NK cells function in colorectal cancer. Cell Death Dis.

[201] Yao J, Li X, Yan L (2019). Role of HGF/c-Met in the treatment of colorectal cancer with liver metastasis. J Biochem Mol Toxicol.

[202] Bradley CA, Dunne PD, Bingham V (2016). Transcriptional upregulation of c-MET is associated with invasion and tumor budding in colorectal cancer. Oncotarget.

[203] Huang C, Ou R, Chen X (2021). Tumor cell-derived SPON2 promotes M2-polarized tumor-associated macrophage infiltration and cancer progression by activating PYK2 in CRC. Journal of Experimental & Clinical Cancer Research.

[204] Eide PW, Moosavi SH, Eilertsen IA (2021). Metastatic heterogeneity of the consensus molecular subtypes of colorectal cancer. NPJ Genom Med.

[205] Liu M, Liu L, Song Y, Li W, Xu L (2022). Targeting macrophages: a novel treatment strategy in solid tumors. J Transl Med.

[206] Valdeolivas A, Amberg B, Giroud N (2024). Profiling the heterogeneity of colorectal cancer consensus molecular subtypes using spatial transcriptomics. NPJ Precis Oncol.

