# Supplementary material for: Macrophages in Colorectal Cancer: from Normal Mucosa to Distant Metastasis: Beyond the M1/M2 Paradigm
Source: J Cancer. 2026 Jan 1;17(1):157–76. doi: 10.7150/jca.126772 (PMC12719567; doi:10.7150/jca.126772)
Supplement: Supplementary file 1 — Supplementary table. [file jcav17p0157s1.pdf]

Table S1

The main markers of macrophages, their biological properties and association with colorectal cancer

Marker, Localization	polarization state	Biological effects	Associations with CRC	References
F4/80 (adhesion G protein-coupled receptor E, EMR1),<br>cell membrane	M0	induction of efferent CD8+ T cells necessary for peripheral tolerance	EMR1 is abnormally expressed in colorectal cancer (CRC) and is a risk factor for liver metastasis (LM) and poor RFS in patients with CRC. Inducing EMR1 in macrophages may promote LNM and CRC progression via JAK2/STAT1,3 signaling upregulation.<br><br>EMR1 is significantly associated with CD68+ CD163+ macrophages, and CRC with a high combined EMR1+CD68+CD163+ score showed worse recurrence-free survival (RFS).	[1, 2, 3]
CCR2 (C-C chemokine receptor type 2 for the chemokine CCL2),<br>cell membrane	M0	involved in the recruitment of monocytes to sites of inflammation	Aberrant expression of CCR2 is associated with negative outcomes in inflammatory bowel disease (IBD), and colon-related metastasis. CCR2 inhibition can reduce the recruitment of myeloid-derived suppressor cells and decrease lung metastasis in breast cancer models Endothelial CCR2 expression has been linked to promoting tumor cell extravasation and pulmonary metastasis, highlighting its significance in cancer progression.	[4, 5, 6]
CD14,<br>cell membrane	M0	co-receptor for bacterial LPS	High CD14 expression in CRC is associated with microsatellite instability, BRAF mutations. High CD14 expression predicts worse outcomes in CRC. High density of CD14+HLA-DR- cells (immature monocytic	[7, 8, 9]<br><br>https://www.proteinatlas.org/ENSG00000170458-CD14

			phenotype) in intraepithelial regions is associated with higher CRC-specific mortality, however, high density of CD14+HLA-DR+ cells (mature monocytic phenotype) in both intraepithelial and stromal regions is associated with lower CRC-specific mortality.	
CD68 (SCARD1- Scavenger Receptor Class D Member 1 GP110, LAMP4), lysosomes, endosomes, cell membrane	M0	A transmembrane glycoprotein that binds to tissue- and organ-specific lectins or selectins, clear cellular debris, promote phagocytosis, and mediate the recruitment and activation of macrophages	CD68+ cells are predominantly found at the invasive front of the tumor compared to the intratumoral area or adjacent normal mucosa. There is a moderate correlation between CD68 and CD163 staining, suggesting that tumors with higher CD68+ infiltration also tend to have higher CD163+ cell infiltration.<br>Prognostic associations in CRC are contradictory.<br>High infiltration of CD68+ TAMs, especially in the tumor stroma, correlates with worse RFS and OS in late-stage CRC patients receiving bevacizumab combined with chemotherapy. Specifically, in stage III CRC, higher infiltration of CD68+ cells in the intratumoral area was associated with reduced overall survival (OS). A high CD206+/CD68+ ratio is associated with improved survival in adjuvant chemotherapy.<br>High CD68+/tumor cell ratio was linked to better survival in 205 CRC patients.	[10-15]<br><br>https://www.proteinatlas.org/ENSG00000129226-CD68<br>https://doi.org/10.1158/1538-7445.AM2022-2530
CSF1R (receptor for colony-stimulating	M0	CSF1R promotes the release of pro-inflammatory chemokines in response to IL-34 and CSF-1, contributing to homeostasis in the colon. CSF1R-expressing	The CSF1/CSF1R axis is essential for the survival and differentiation of M2 tumor associated macrophages (TAM) in CRC. CSF1R expression is enriched in TAMs within CRC tumors, and its expression in	[16]

factor 1, CD115), cell membrane		macrophages are involved in tissue repair processes in the colon.	macrophages is associated with poor prognosis in CRC patients.	https://www.ncbi.nlm.nih.gov/gene/1436proteinatlas.org/ENSG00000182578-CSF1R
Ly6C1 (lymphocyte antigen 6 family member C1), cell membrane	M0	Ly6C1, a member of the Ly-6 superfamily, plays a crucial role in immune responses and cell signaling. Ly6C1 is anchored to the cell membrane through a glycosyl-phosphatidylinositol (GPI) lipid anchor, allowing them to localize to specific membrane domains called lipid rafts. Cross-linking of Ly-6 proteins like Ly-6A/Sca-1 can trigger simultaneous stimulatory and inhibitory responses in cells, leading to cytokine production, growth inhibition, and apoptosis. Additionally, Ly6C1 may be involved in regulating complement activation and pathogen clearance, highlighting its importance in host defense mechanisms.	Ly6C-high macrophages contribute to tumor initiation and malignant progression in CRC. Participate in the creation of a pre-metastatic niche and subsequent colonization of metastatic sites by tumor cells.	[17, 18] Klikněte nebo klepněte sem a zadejte text.
PPARG (Peroxisome Proliferator-Activated Receptor Gamma),	M0	a type II nuclear receptor, a transcription factor. PPARG is an important regulator of macrophage polarization, with PPARG activation driving the M2 phenotype through	High PPARG expression in tumors was not significantly associated with worse prognosis in CRC patients, as indicated by a study analyzing PPARG gene expression in CRC tumors and adjacent normal tissues. However, in lung adenocarcinoma, low PPARG	[19-23] https://www.proteinatlas.org/ENSG00000132170-PPARG

nucleus, cytoplasm		upregulation of Arg1 and Mgl1 genes.	expression was linked to poor prognosis. The study on hypopharyngeal squamous cell carcinoma (HSCC) revealed that PPARG expression variations were significantly associated with the tumor node metastasis (TNM). Role of PPARG expression in cancer prognosis and metastasis can vary depending on the specific cancer type.	
CX3CR1 (C-X3-C Motif Chemokine Receptor 1 for CX3CL1), cell membrane	M0	Involved in the adhesion and migration of monocytes, macrophages, and other immune cells	CX3CR1 expression is significantly elevated in poorly differentiated CRC compared to moderate-well-differentiated tumors. Higher CX3CR1 expression is associated with advanced clinical stages, metastasis, and recurrence within 3 years.	[24, 25]
ITGAM (Integrin alpha M subunit, CD11b), cell membrane	M0	Cell adhesion, migration, and phagocytosis	The concentration of ITGAM-positive exosomes is lower in both primary CRC and metastatic CRC compared to the healthy control. ITGAM expression is highest in healthy control, followed by colonic adenomas, and lowest in primary CRC and CRC with hepatic metastases.	[26, 27] https://www.proteinatlas.org/ENSG00000169896-ITGAM
FCGRIA (**Fc** Gamma Receptor Ia, CD64), cell membrane	M1	**antibody-dependent cell-mediated cytotoxicity (ADCC), antibody-dependent cellular phagocytosis (ADCP)**	High infiltration of CD64+ macrophages in CRC, particularly at the tumor front, is associated with improved patient survival. Mediates ADCC by macrophages against tumor cells.	[28, 29] Klikněte nebo klepněte sem a zadejte text.
CD80, cell membrane	M1	CD80 is an inducible co-stimulatory molecule on APC. Interacts with CD28 and CTLA-4 on T cells to regulate T cell activation and tolerance.	CD80+ macrophages are more prevalent in less invasive T1 tumors compared to more advanced stages. CD80+ macrophages, although present at low numbers, are associated with better prognosis in CRC.	[30-31] https://www.proteinatlas.org/ENSG00000121594-CD80

			In stage III colorectal tumors, a lower CD80/CD163 ratio is associated with decreased OS.	
CD86,<br>cell membrane	M1,<br>M2b	co-stimulatory molecule related to CD80	Lower infiltration of CD86+ macrophages is associated with more advanced tumor stages and higher rates of tumor recurrence and mortality. Stage II-III CRC patients with a low CD86/CD163 ratio had shorter RFS and OS.	[32-34]<br>https://www.proteinatlas.org/ENSG00000114013-CD86
CD40,<br>cell membrane, secreted	M1	type I transmembrane protein on antigen-presenting cells and is required for their activation. Promotes pro-inflammatory and anti-tumor responses when activated on macrophages	High CD40 expression in CRC tissues is associated with better OS and DFS.	[30, 35]<br>proteinatlas.org/ENSG00000101017-CD40
iNOS,<br>cytoplasm	M1,<br>M2d	Produces nitric oxide (NO) with a cytotoxic activities against pathogens and tumor cells	M1 macrophages, which express iNOS, have anti-tumor effects.<br>In a study of 205 CRC patients, iNOS+ macrophages did not demonstrate any significant benefit to patient outcomes.<br>Also, infiltration of CD68+/iNOS TAMs in the tumor stroma is a negative prognostic factor.	[10, 36-39]<br>Klikněte nebo klepněte sem a zadejte text.
MHC-II,<br>cell membrane	M1,<br>M2a<br>M2b	antigen presentation to CD4+ T cells	In primary CRC, increased MHC-II expression is associated with increased tumor-infiltrating lymphocytes and improved prognosis. Low MHC-II expression may reflect poor interactions between antigen-presenting cells (APC) and helper T-cell and reduced cytotoxic T lymphocytes mediated anti-tumor activity.	[40-43]<br>Klikněte nebo klepněte sem a zadejte text.
TLR2<br>TLR4 (Toll-Like Receptor 2 and 4), cell	M1, M2c	Recognize a wide range of pathogen-associated molecular patterns (PAMPs) from gram-	Among stage III patients a strong TLR2 expression associates with a better prognosis. Among patients with stage II CRC, a strong TLR4 expression associate with a worse DSS.	[30, 44-46]<br>https://www.proteinatlas.org/search/TLR2

membrane, cytoplasm		positive bacteria, mycobacteria, fungi, and viruses		
IL1R1 (Interleukin-1 Receptor Type 1), cell membrane	M1<br>M2b	receptor for IL-1α and IL-1ß, pro-inflammatory cytokines that initiate inflammatory responses and mediate innate immunity against pathogens	Patients with progressive CRC present higher levels of IL-1R1 in the pCRC tissue than patients responsive to the therapy or with a stable disease.<br>IL-1R1 this is a maker of poor prognosis in CRC	[47, 48]
IL-10R (Interleukin-10 Receptor), cell membrane	M1<br>M2b	IL-10R signaling helps maintain immune homeostasis by suppressing excessive inflammatory responses, as IL-10 is a crucial immunosuppressive agent. Also, IL-10R signaling can modulate the expression of MHC class II and co-stimulatory molecules, affecting antigen presentation by macrophage	Elevated IL-10 levels within the tumor microenvironment or in the systemic circulation contribute to an immunosuppressive milieu in colorectal cancer by dampening antigen presentation, reducing cytotoxic T-cell and NK-cell activity, and promoting tumor cell proliferation and chemoresistance, all of which are associated with unfavorable clinical outcomes . Activation of the IL-10 receptor complex (IL-10RA/IL-10RB) further enhances STAT3- and NF-κB-dependent signaling pathways, supporting tumor cell survival, epithelial to mesenchymal transition, and metastatic progression in CRC . Consequently, increased IL-10 expression or IL-10R activation is considered a negative prognostic indicator and a potential therapeutic target in CRC.	[49-52]<br>https://www.proteinatlas.org/search/IL-10R+
CD163,<br>cell membrane	M2a,<br>M2c	Scavenger receptor involved in clearance of hemoglobin-haptoglobin complexes and anti-inflammatory functions	High levels of CD163 expression in serum and tumor tissues have been associated with a worse prognosis. High expression levels correlate with lower OS rates.	[53-55]<br>https://www.proteinatlas.org/ENSG00000177575-CD163

CD206 (Mannose Receptor), cell membrane	M2a, M2c	a C-type lectin, Involved in pathogen recognition and tissue remodeling	Higher density of CD206+ macrophages is associated with poorer prognosis in CRC. A high ratio of CD206+/CD68+ macrophages is significantly associated with poor survival in stage II CRC patients. Adjuvant chemotherapy significantly improved RFS and OS for patients with a high CD206+/CD68+ ratio of TAMs.	[10, 33, 56] https://www.proteinatlas.org/ENSG00000260314-MRC1/pathology
CLEC7A (CD301) (Dectin-1), cell membrane	M2a	C-type lectin receptor, is a key innate immune receptor involved in coordinating host defense against fungi. It recognizes β-1,3-glucan, a major structural component of fungal cell walls, induces anti-fungal responses.	CLEC7A promotes pro-tumor functions of macrophages. CLEC7A promotes tumor progression by regulating the immune microenvironment. Depletion of Clec7a in macrophages in vivo increases the infiltration of tumor tissue by CD4+ and CD8+ T-cells. An analysis of the literature did not demonstrate the role of CLEC7A as a prognostic marker in CRC.	[57, 58] https://www.proteinatlas.org/ENSG00000172243-CLEC7A
CD36, cell membrane	M2a	scavenger receptor that mediates the uptake of oxidized lipids and apoptotic cells. Promotes inflammatory responses and phagocytosis. CD36 may interact with other receptors, such as integrins, TLRs, or tetraspanins.	High CD36 mRNA levels are associated with reduced 5-year survival in CRC patients. CD36 expression is highest in macrophages in the liver, particularly in metastasis-associated macrophages (MAMs) within metastatic liver tumors	[59-61] https://www.proteinatlas.org/ENSG00000135218-CD36/pathology/colorectal+cancer
CD209 (DC-SIGN)(Dendritic Cell-Specific Intercellular adhesion	M2a, 2b, 2c, 2d	a C-type lectin receptor is present on the surface of both macrophages and dendritic cells. Involved in antigen uptake and presentation.	Expression is increased in metastatic CRC cell lines and patient tissues. Higher DC-SIGN expression is associated with reduced OS in CRC patients.	[62-64]

molecule-3-Grabbing Non-integrin), cell membrane			DC-SIGN facilitates CRC metastasis both in vitro and in vivo. It forms a complex with Lyn and p85, promoting metastasis by increasing PI3K/Akt/β-catenin signaling.<br><br>Soluble DC-SIGN (sDC-SIGN) levels in serum are significantly higher in CRC patients with distant metastasis compared to non-metastatic patients.	
FCGR3A (Fc Gamma Receptor IIIa, low ffinity Fc receptor, CD16), cell membrane, secreted	M2a, 2c	mediates antibody-dependent cell-mediated cytotoxicity (ADCC) and antibody-dependent phagocytosis (ADP)	Genetic polymorphisms in FcγRIIIa have been associated with response to anti-EGFR antibody therapy in metastatic CRC patients. Patients carrying the FcγRIIIa-158F/F genotype tend to had a less favorable prognosis compared to those with V/V or V/F genotypes. Survival analysis indicated that FCGR3A serves as a prognostic risk factor in most types of cancer.	[65-66] Klikněte nebo klepněte sem a zadejte text.
MSR1 (CD204) cell membrane	M2a, 2b, 2c, 2d	a scavenger receptor that recognizes and clears modified lipoproteins and bacterial products.	Higher infiltration of CD204-positive macrophages into colorectal tumors is associated with shorter OS and RFS in patients with stage II and III CRC.<br>Also, in vitro studies have shown that M2-polarized macrophages with high CD204 expression enhance the proliferation and invasion of CRC cell lines.	[67-69]

TIMD4 TIMD4 (T-cell immunoglobulin and mucin domain containing 4), cell membrane	M2a	a phosphatidylserine receptor involved in recognition and clearance of apoptotic cells.	TIMD4 expression on macrophages may serve as a marker for a subset of tissue-resident macrophages with immunosuppressive functions in CRC. The presence of TIMD4-positive macrophages in CRC is associated with worse clinical outcomes, including increased tumor growth and metastasis.	[70, 71]
CLEC10A (C-type lectin domain family 10 member A receptor, CD301), cell membrane	M2a	recognizes and binds to various glycan structures.	CLEC10A expression has been correlated with clinical outcomes in several cancers, including CRC. Higher expression levels of CLEC10A ligands on tumor cells have been associated with poorer disease-free survival in stage III CRC patients. The presence of CLEC10A-positive macrophages in the tumor microenvironment may indicate a more aggressive tumor phenotype and poorer prognosis.	[72-74]
FIZZ1 (RELM-α) (RELM-α) (Resistin-Like Molecule Alpha or RELM-α), cell membrane	M2a	FIZZ1 is a resistin-like molecule involved in wound healing and tissue repair processes.	Some studies have explored the presence of FIZZ1 in stool samples from American patients with CRC, suggesting its potential as a biomarker for CRC detection.<br>No prognostic associations in CRC have been established yet.	[75-78]
Arg1 (Arginase-1), cytoplasm	M2a	An enzyme involved in the urea cycle, catalyzing the conversion of L-arginine to L-ornithine and urea.	The expression levels of Arg-1 is significantly higher in CRC compared to the corresponding normal colon tissues. Increased Arg-1 expression is associated with stage III-IV tumors. Arg-1 overexpression was associated with shorter OS and DFS in advanced CRC stages (III + IV) , but not at early stages (I + II) in multivariate analysis.	[79-81]<br>https://www.proteinatlas.org/ENSG00000118520-ARG1

			The activation of ARG1 is also associated with the migration ability and metastatic colonization of colon cancer cells, and blocking this process may be a novel strategy for controlling malignancies.	
CD155 (poliovirus receptor (PVR), cell membrane, secreted	M2a	-	Macrophages in the CRC tissue express high levels of CD155 compared to those from adjacent normal tissues. The expression level of macrophage CD155 was higher in stage III/IV CRC compared to stage I/II and was negatively associated with the survival of CRC patients. Additionally, CD155+ macrophages promote migration, invasion, and growth of CRC cells.	[82, 83] https://www.proteinatlas.org/ENSG00000073008-PVR
VEGF (Vascular Endothelial Growth Factor), VEGFR1 (VEGF receptor), cell membrane	M2d	VEGF stimulates endothelial cell proliferation, migration, and survival, facilitating the formation of new blood vessels. The VEGF receptor (VEGFR1) is a family of three closely related, membrane-spanning peptides containing seven extracellular immunoglobulin-like domains and two intracellular tyrosine kinases. Binding of VEGF to VEGFR1 stimulates endothelial cell migration, and may mediate vascular organization.	VEGF overexpression in CRC is associated with poor OS. While VEGFR1 expression in primary CRC tumor patients did not predict prognosis; high percentage of VEGFR1+ cells in liver metastasis was associated with worse patient outcome. VEGFR1+ metastasis-associated macrophages contribute to metastasis in CRC and were identified as a potential new prognostic marker for disease recurrence.	[84-86] https://www.proteinatlas.org/ENSG00000112715-VEGFA/pathology
SIGLEC1 (CD169), Sialoadhesin,	Non M1/M2	Cell adhesion molecule. Antigen presentation and the modulation of T-cell responses	A high density of CD169+ macrophages in RLNs is significantly associated with longer OS in CRC patients. CD169+ cells to CD68+	[87-90]

Plasmatic membrane, intracellular			cells ratio in RLNs was an independent prognostic factor for CRC. The number of CD169+ sinus macrophages in regional lymph node (RLN) decreased in CRC patients with lymph nodes metastasis. Also, the density of CD169+ macrophages in RLNs positively correlates with the number of CD8+ cytotoxic T cells infiltrating tumor tissues. CD169+ macrophages in RLNs are thought to promote CD8+ T-cell-mediated antitumor immunity, contributing to a better prognosis for CRC patients.<br>In primary CRC, CD169 macrophages can exhibit protumor effects.	https://www.proteinatlas.org/ENSG00000088827-SIGLEC1/pathology
CD63, membranes of intracellular vesicles (constituitive), cell membrane (inducible)	Non M1/M2	CD63 is a member of the tetraspanin superfamily of activation-linked cell surface antigens. Regulates phagocytosis, antigen presentation, and secretion of inflammatory mediators	High CD63 expression in CRC is associated with advanced stages of the disease, poor differentiation, mucinous histology and EMT-associated secretory phenotype. It predicts an unfavorable prognosis in CRC patients, including those with metastatic disease in pCRC. CD63 immunohistochemistry can be used to identify patients with an increased risk of recurrence who might benefit from adjuvant therapy.	[91-93]<br>https://www.proteinatlas.org/ENSG00000135404-CD63/pathology

Abbreviations: APC: antigen-presenting cells; CRC: colorectal cancer; IBD: inflamatory bowel desease; LN: lymph node; LM: lymph metastasis; MAM: metastasis-associated macrophages; OS: overall survival; RLNs; regional lymph nodes; RFS: recurence free survival; TAMs: tumor-associated macrophages; TNM: tumor node metastasis.

References

1. Akter R, Park R, Lee SK, et al. Upregulation of EMR1 (ADGRE1) by Tumor-Associated Macrophages Promotes Colon Cancer Progression by Activating the JAK2/STAT1,3 Signaling Pathway in Tumor Cells. *Int J Mol Sci*. 2024;25(8):4388. doi:10.3390/ijms25084388

2. Qiao T, Yang W, He X, et al. Dynamic differentiation of F4/80+ tumor-associated macrophage and its role in tumor vascularization in a syngeneic mouse model of colorectal liver metastasis. *Cell Death Dis*. 2023;14(2):117. doi:10.1038/s41419-023-05626-1

3. Shin AE, Tesfagiorgis Y, Larsen F, et al. F4/80+Ly6Chigh Macrophages Lead to Cell Plasticity and Cancer Initiation in Colitis. *Gastroenterology*. 2023;164(4):593-609.e13. doi:10.1053/j.gastro.2023.01.002

4. Yuan J. CCR2: A characteristic chemokine receptor in normal and pathological intestine. *Cytokine*. 2023;169:156292. doi:10.1016/j.cyto.2023.156292

5. Roblek M, Protsyuk D, Becker PF, et al. CCL2 Is a Vascular Permeability Factor Inducing CCR2-Dependent Endothelial Retraction during Lung Metastasis. *Molecular Cancer Research*. 2019;17(3):783-793. doi:10.1158/1541-7786.MCR-18-0530

6. Hao Q, Vadgama J V., Wang P. CCL2/CCR2 signaling in cancer pathogenesis. *Cell Communication and Signaling*. 2020;18(1):82. doi:10.1186/s12964-020-00589-8

7. Chen D, Wang H. The clinical and immune features of CD14 in colorectal cancer identified via large-scale analysis. *Int Immunopharmacol*. 2020;88:106966. doi:10.1016/j.intimp.2020.106966

8. Väyrynen JP, Haruki K, Väyrynen SA, et al. Prognostic significance of myeloid immune cells and their spatial distribution in the colorectal cancer microenvironment. *J Immunother Cancer*. 2021;9(4):e002297. doi:10.1136/jitc-2020-002297

9. Simmons D, Tan S, Tenen D, Nicholson-Weller A, Seed B. Monocyte antigen CD14 is a phospholipid anchored membrane protein. *Blood*. 1989;73(1):284-289. doi:10.1182/blood.V73.1.284.284

10. Wang H, Tian T, Zhang J. Tumor-Associated Macrophages (TAMs) in Colorectal Cancer (CRC): From Mechanism to Therapy and Prognosis. *Int J Mol Sci*. 2021;22(16):8470. doi:10.3390/ijms22168470

11. Pinto ML, Rios E, Durães C, et al. The Two Faces of Tumor-Associated Macrophages and Their Clinical Significance in Colorectal Cancer. *Front Immunol*. 2019;10. doi:10.3389/fimmu.2019.01875

12. Zhang M, He Y, Sun X, et al. A high M1/M2 ratio of tumor-associated macrophages is associated with extended survival in ovarian cancer patients. *J Ovarian Res*. 2014;7(1):19. doi:10.1186/1757-2215-7-19

13. Feng Q, Chang W, Mao Y, et al. Tumor-associated Macrophages as Prognostic and Predictive Biomarkers for Postoperative Adjuvant Chemotherapy in Patients with Stage II Colon Cancer. *Clinical Cancer Research*. 2019;25(13):3896-3907. doi:10.1158/1078-0432.CCR-18-2076

14. Inagaki K, Kunisho S, Takigawa H, et al. Role of tumor-associated macrophages at the invasive front in human colorectal cancer progression. *Cancer Sci*. 2021;112(7):2692-2704. doi:10.1111/cas.14940

15. Zhang J, Li S, Liu F, Yang K. Role of CD68 in tumor immunity and prognosis prediction in pan-cancer. *Sci Rep*. 2022;12(1):7844. doi:10.1038/s41598-022-11503-2

16. Zhu M, Bai L, Liu X, et al. Silence of a dependence receptor CSF1R in colorectal cancer cells activates tumor-associated macrophages. *J Immunother Cancer*. 2022;10(12):e005610. doi:10.1136/jitc-2022-005610

17. Nair R, Lannagan TRM, Jackstadt R, et al. Co-inhibition of TGF-β and PD-L1 pathways in a metastatic colorectal cancer mouse model triggers interferon responses, innate cells and T cells, alongside metabolic changes and tumor resistance. *Oncoimmunology*. 2024;13(1). doi:10.1080/2162402X.2024.2330194

18. Du X, Gu H, Sun Y, Hu Y. Ly-6D of Japanese flounder (Paralichthys olivaceus) functions as a complement regulator and promotes host clearance of pathogen. *Dev Comp Immunol*. 2021;122:104104. doi:10.1016/j.dci.2021.104104

19. Schöckel L, Woischke C, Surendran SA, et al. PPARG activation promotes the proliferation of colorectal cancer cell lines and enhances the antiproliferative effect of 5-fluorouracil. *BMC Cancer*. 2024;24(1):234. doi:10.1186/s12885-024-11985-5

20. Ogino S, Shima K, Baba Y, et al. Colorectal Cancer Expression of Peroxisome Proliferator-Activated Receptor γ (PPARG, PPARgamma) Is Associated With Good Prognosis. *Gastroenterology*. 2009;136(4):1242-1250. doi:10.1053/j.gastro.2008.12.048

21. Hernandez-Quiles M, Broekema MF, Kalkhoven E. PPARgamma in Metabolism, Immunity, and Cancer: Unified and Diverse Mechanisms of Action. *Front Endocrinol (Lausanne)*. 2021;12. doi:10.3389/fendo.2021.624112

22. Villa ALP, Parra RS, Feitosa MR, et al. PPARG expression in colorectal cancer and its association with staging and clinical evolution. *Acta Cir Bras*. 2020;35(7). doi:10.1590/s0102-865020200070000008

23. Zhao M, Li X, Zhang Y, Zhu H, Han Z, Kang Y. PPARG Drives Molecular Networks as an Inhibitor for the Pathologic Development and Progression of Lung Adenocarcinoma. *PPAR Res*. 2020;2020:1-7. doi:10.1155/2020/6287468

24. Zheng J, Yang M, Shao J, Miao Y, Han J, Du J. Chemokine receptor CX3CR1 contributes to macrophage survival in tumor metastasis. *Mol Cancer*. 2013;12(1):141. doi:10.1186/1476-4598-12-141

25. Yue Y, Zhang Q, Sun Z. CX3CR1 Acts as a Protective Biomarker in the Tumor Microenvironment of Colorectal Cancer. *Front Immunol*. 2022;12. doi:10.3389/fimmu.2021.758040

26. Guo W, Cai Y, Liu X, et al. Single-Exosome Profiling Identifies ITGB3+ and ITGAM+ Exosome Subpopulations as Promising Early Diagnostic Biomarkers and Therapeutic Targets for Colorectal Cancer. *Research*. 2023;6. doi:10.34133/research.0041

27. Schmid MC, Khan SQ, Kaneda MM, et al. Integrin CD11b activation drives anti-tumor innate immunity. *Nat Commun*. 2018;9(1):5379. doi:10.1038/s41467-018-07387-4

28. Norton SE, Dunn ETJ, McCall JL, Munro F, Kemp RA. Gut macrophage phenotype is dependent on the tumor microenvironment in colorectal cancer. *Clin Transl Immunology*. 2016;5(4). doi:10.1038/cti.2016.21

29. Wu J, Li Y, Rendahl A, Bhargava M. Novel Human FCGR1A Variants Affect CD64 Functions and Are Risk Factors for Sarcoidosis. *Front Immunol*. 2022;13. doi:10.3389/fimmu.2022.841099

30. Mola S, Pandolfo C, Sica A, Porta C. The Macrophages-Microbiota Interplay in Colorectal Cancer (CRC)-Related Inflammation: Prognostic and Therapeutic Significance. *Int J Mol Sci*. 2020;21(18):6866. doi:10.3390/ijms21186866

31. Subauste CS, de Waal Malefyt R, Fuh F. Role of CD80 (B7.1) and CD86 (B7.2) in the Immune Response to an Intracellular Pathogen. *The Journal of Immunology*. 1998;160(4):1831-1840. doi:10.4049/jimmunol.160.4.1831

32. Xu G, Jiang L, Ye C, et al. The Ratio of CD86+/CD163+ Macrophages Predicts Postoperative Recurrence in Stage II-III Colorectal Cancer. *Front Immunol*. 2021;12. doi:10.3389/fimmu.2021.724429

33. Xu G, Mo Y, Li J, Wei Q, Zhou F, Chen J. Two tripartite classification systems of CD86+ and CD206+ macrophages are significantly associated with tumor recurrence in stage II-III colorectal cancer. *Front Immunol*. 2023;14. doi:10.3389/fimmu.2023.1136875

34. Zhang Q, Ma R, Chen H, et al. CD86 Is Associated with Immune Infiltration and Immunotherapy Signatures in AML and Promotes Its Progression. *J Oncol*. 2023;2023:1-19. doi:10.1155/2023/9988405

35. Kinouchi M, Miura K, Mizoi T, et al. Infiltration of CD40-Positive Tumor-Associated Macrophages Indicates a Favorable Prognosis in Colorectal Cancer Patients. *Hepatogastroenterology*. Published online May 14, 2012. doi:10.5754/hge12372

36. Edin S, Wikberg ML, Dahlin AM, et al. The Distribution of Macrophages with a M1 or M2 Phenotype in Relation to Prognosis and the Molecular Characteristics of Colorectal Cancer. *PLoS One*. 2012;7(10):e47045. doi:10.1371/journal.pone.0047045

37. Lu L, Liu YJ, Cheng PQ, Dan H, Xu HC, Ji G. Macrophages play a role in inflammatory transformation of colorectal cancer. *World J Gastrointest Oncol*. 2021;13(12):2013-2028. doi:10.4251/wjgo.v13.i12.2013

38. Xue Q, Yan Y, Zhang R, Xiong H. Regulation of iNOS on Immune Cells and Its Role in Diseases. *Int J Mol Sci*. 2018;19(12):3805. doi:10.3390/ijms19123805

39. Kashfi K, Kannikal J, Nath N. Macrophage Reprogramming and Cancer Therapeutics: Role of iNOS-Derived NO. *Cells*. 2021;10(11):3194. doi:10.3390/cells10113194

40. Griffith BD, Turcotte S, Lazarus J, et al. MHC Class II Expression Influences the Composition and Distribution of Immune Cells in the Metastatic Colorectal Cancer Microenvironment. *Cancers (Basel)*. 2022;14(17):4092. doi:10.3390/cancers14174092

41. Coletta S, Lonardi S, Sensi F, et al. Tumor Cells and the Extracellular Matrix Dictate the Pro-Tumoral Profile of Macrophages in CRC. *Cancers (Basel)*. 2021;13(20):5199. doi:10.3390/cancers13205199

42. Guillaume J, Leufgen A, Hager FT, Pabst O, Cerovic V. MHCII expression on gut macrophages supports T cell homeostasis and is regulated by microbiota and ontogeny. *Sci Rep*. 2023;13(1):1509. doi:10.1038/s41598-023-28554-8

43. Hou S, Zhao Y, Chen J, Lin Y, Qi X. Tumor-associated macrophages in colorectal cancer metastasis: molecular insights and translational perspectives. *J Transl Med*. 2024;22(1):62. doi:10.1186/s12967-024-04856-x

44. Beilmann-Lehtonen I, Böckelman C, Mustonen H, Koskensalo S, Hagström J, Haglund C. The prognostic role of tissue TLR2 and TLR4 in colorectal cancer. *Virchows Archiv*. 2020;477(5):705-715. doi:10.1007/s00428-020-02833-5

45. Nihon-Yanagi Y, Terai K, Murano T, Matsumoto T, Okazumi S. Tissue expression of Toll-like receptors 2 and 4 in sporadic human colorectal cancer. *Cancer Immunology, Immunotherapy*. 2012;61(1):71-77. doi:10.1007/s00262-011-1085-4

46. Hu X, Fatima S, Chen M, et al. Toll-like receptor 4 is a master regulator for colorectal cancer growth under high-fat diet by programming cancer metabolism. *Cell Death Dis*. 2021;12(8):791. doi:10.1038/s41419-021-04076-x

47. Gelfo V, Mazzeschi M, Grilli G, et al. A Novel Role for the Interleukin-1 Receptor Axis in Resistance to Anti-EGFR Therapy. *Cancers (Basel)*. 2018;10(10):355. doi:10.3390/cancers10100355

48. Koncina E, Nurmik M, Pozdeev VI, et al. IL1R1+ cancer-associated fibroblasts drive tumor development and immunosuppression in colorectal cancer. *Nat Commun*. 2023;14(1):4251. doi:10.1038/s41467-023-39953-w

49. Li J, Huang L, Zhao H, Yan Y, Lu J. The Role of Interleukins in Colorectal Cancer. *Int J Biol Sci*. 2020;16(13):2323-2339. doi:10.7150/ijbs.46651

50. Rossowska J, Anger N, Szczygieł A, Mierzejewska J, Pajtasz-Piasecka E. Reprogramming the murine colon cancer microenvironment using lentivectors encoding shRNA against IL-10 as a component of a potent DC-based chemoimmunotherapy. *Journal of Experimental & Clinical Cancer Research*. 2018;37(1):126. doi:10.1186/s13046-018-0799-y

51. del Carmen S, de Moreno de LeBlanc A, Levit R, et al. Anti-cancer effect of lactic acid bacteria expressing antioxidant enzymes or IL-10 in a colorectal cancer mouse model. *Int Immunopharmacol*. 2017;42:122-129. doi:10.1016/j.intimp.2016.11.017

52. de Araujo Junior RF, Eich C, Jorquera C, et al. Ceramide and palmitic acid inhibit macrophage-mediated epithelial–mesenchymal transition in colorectal cancer. *Mol Cell Biochem*. 2020;468(1-2):153-168. doi:10.1007/s11010-020-03719-5

53. Xue T, Yan K, Cai Y, et al. Prognostic significance of CD163+ tumor-associated macrophages in colorectal cancer. *World J Surg Oncol*. 2021;19(1):186. doi:10.1186/s12957-021-02299-y

54. Ma S, Zhao Y, Liu X, et al. CD163 as a Potential Biomarker in Colorectal Cancer for Tumor Microenvironment and Cancer Prognosis: A Swedish Study from Tissue Microarrays to Big Data Analyses. *Cancers (Basel)*. 2022;14(24):6166. doi:10.3390/cancers14246166

55. Krijgsman D, De Vries NL, Andersen MN, et al. CD163 as a Biomarker in Colorectal Cancer: The Expression on Circulating Monocytes and Tumor-Associated Macrophages, and the Soluble Form in the Blood. *Int J Mol Sci*. 2020;21(16):5925. doi:10.3390/ijms21165925

56. Yin Y, Liu B, Cao Y, et al. Colorectal Cancer-Derived Small Extracellular Vesicles Promote Tumor Immune Evasion by Upregulating PD-L1 Expression in Tumor-Associated Macrophages. *Advanced Science*. 2022;9(9). doi:10.1002/advs.202102620

57. Liu X, Lv K, Wang J, et al. C-type lectin receptor Dectin-1 blockade on tumour-associated macrophages improves anti-PD-1 efficacy in gastric cancer. *Br J Cancer*. 2023;129(4):721-732. doi:10.1038/s41416-023-02336-5

58. Tang C, Sun H, Kadoki M, et al. Blocking Dectin-1 prevents colorectal tumorigenesis by suppressing prostaglandin E2 production in myeloid-derived suppressor cells and enhancing IL-22 binding protein expression. *Nat Commun*. 2023;14(1):1493. doi:10.1038/s41467-023-37229-x

59. Yang P, Qin H, Li Y, et al. CD36-mediated metabolic crosstalk between tumor cells and macrophages affects liver metastasis. *Nat Commun*. 2022;13(1):5782. doi:10.1038/s41467-022-33349-y

60. Guerrero-Rodríguez SL, Mata-Cruz C, Pérez-Tapia SM, Velasco-Velázquez MA. Role of CD36 in cancer progression, stemness, and targeting. *Front Cell Dev Biol*. 2022;10. doi:10.3389/fcell.2022.1079076

61. Zaidi NE, Shazali NAH, Leow TC, et al. CD36-Fatty Acid-Mediated Metastasis via the Bidirectional Interactions of Cancer Cells and Macrophages. *Cells*. 2022;11(22):3556. doi:10.3390/cells11223556

62. Yuan M, Zhang X, Zhang J, et al. DC-SIGN–LEF1/TCF1–miR-185 feedback loop promotes colorectal cancer invasion and metastasis. *Cell Death Differ*. 2020;27(1):379-395. doi:10.1038/s41418-019-0361-2

63. Jiang Y, Zhang C, Chen K, et al. The Clinical Significance of DC-SIGN and DC-SIGNR, which Are Novel Markers Expressed in Human Colon Cancer. *PLoS One*. 2014;9(12):e114748. doi:10.1371/journal.pone.0114748

64. McGreal EP, Miller JL, Gordon S. Ligand recognition by antigen-presenting cell C-type lectin receptors. *Curr Opin Immunol*. 2005;17(1):18-24. doi:10.1016/j.coi.2004.12.001

65. Calemma R, Ottaiano A, Trotta AM, et al. Fc gamma receptor IIIa polymorphisms in advanced colorectal cancer patients correlated with response to anti-EGFR antibodies and clinical outcome. *J Transl Med*. 2012;10(1):232. doi:10.1186/1479-5876-10-232

66. Li L, Huang Z, Du K, et al. Integrative Pan-Cancer Analysis Confirmed that FCGR3A is a Candidate Biomarker Associated With Tumor Immunity. *Front Pharmacol*. 2022;13. doi:10.3389/fphar.2022.900699

67. TADA Y, MATSUMI Y, HARA K, et al. Infiltration of CD204-overexpressing Macrophages Contributes to the Progression of Stage II and III Colorectal Cancer. *Anticancer Res*. 2021;41(10):4857-4865. doi:10.21873/anticanres.15299

68. Kazakova E, Iamshchikov P, Larionova I, Kzhyshkowska J. Macrophage scavenger receptors: Tumor support and tumor inhibition. *Front Oncol*. 2023;12. doi:10.3389/fonc.2022.1096897

69. Gudgeon J, Marín-Rubio JL, Trost M. The role of macrophage scavenger receptor 1 (MSR1) in inflammatory disorders and cancer. *Front Immunol*. 2022;13. doi:10.3389/fimmu.2022.1012002

70. Chow A, Schad S, Green MD, et al. Tim-4+ cavity-resident macrophages impair anti-tumor CD8+ T cell immunity. *Cancer Cell*. 2021;39(7):973-988.e9. doi:10.1016/j.ccell.2021.05.006

71. Wang Z, Chen C, Su Y, Ke N. Function and characteristics of TIM-4 in immune regulation and disease (Review). *Int J Mol Med*. 2022;51(2):10. doi:10.3892/ijmm.2022.5213

72. Qin Y, Wang L, Zhang L, et al. Immunological role and prognostic potential of CLEC10A in pan-cancer. *Am J Transl Res*. 2022;14(5):2844-2860.

73. Szczykutowicz J. Ligand Recognition by the Macrophage Galactose-Type C-Type Lectin: Self or Non-Self?—A Way to Trick the Host's Immune System. *Int J Mol Sci*. 2023;24(23):17078. doi:10.3390/ijms242317078

74. Heger L, Balk S, Lühr JJ, et al. CLEC10A Is a Specific Marker for Human CD1c+ Dendritic Cells and Enhances Their Toll-Like Receptor 7/8-Induced Cytokine Secretion. *Front Immunol*. 2018;9. doi:10.3389/fimmu.2018.00744

75. Scheurlen KM, Billeter AT, O'Brien SJ, Galandiuk S. Metabolic dysfunction and early-onset colorectal cancer – how macrophages build the bridge. *Cancer Med*. 2020;9(18):6679-6693. doi:10.1002/cam4.3315

76. Órdenes P, Carril Pardo C, Elizondo-Vega R, Oyarce K. Current Research on Molecular Biomarkers for Colorectal Cancer in Stool Samples. *Biology (Basel)*. 2023;13(1):15. doi:10.3390/biology13010015

77. Shi Y, Zhu N, Qiu Y, et al. Resistin-like molecules: a marker, mediator and therapeutic target for multiple diseases. *Cell Communication and Signaling*. 2023;21(1):18. doi:10.1186/s12964-022-01032-w

78. Aras S, Zaidi MR. TAMeless traitors: macrophages in cancer progression and metastasis. *Br J Cancer*. 2017;117(11):1583-1591. doi:10.1038/bjc.2017.356

79. Ma Z, Lian J, Yang M, et al. Overexpression of Arginase-1 is an indicator of poor prognosis in patients with colorectal cancer. *Pathol Res Pract*. 2019;215(6):152383. doi:10.1016/j.prp.2019.03.012

80. Wang X, Xiang H, Toyoshima Y, et al. Arginase-1 inhibition reduces migration ability and metastatic colonization of colon cancer cells. *Cancer Metab*. 2023;11(1):1. doi:10.1186/s40170-022-00301-z

81. Ma RY, Black A, Qian BZ. Macrophage diversity in cancer revisited in the era of single-cell omics. *Trends Immunol*. 2022;43(7):546-563. doi:10.1016/j.it.2022.04.008

82. Zhu X, Liang R, Lan T, et al. Tumor-associated macrophage-specific CD155 contributes to M2-phenotype transition, immunosuppression, and tumor progression in colorectal cancer. *J Immunother Cancer*. 2022;10(9):e004219. doi:10.1136/jitc-2021-004219

83. Paolini R, Molfetta R. CD155 and Its Receptors as Targets for Cancer Therapy. *Int J Mol Sci*. 2023;24(16):12958. doi:10.3390/ijms241612958

84. Mazeda I, Martins SF, Garcia EA, Rodrigues M, Longatto A. VEGF Expression in Colorectal Cancer Metastatic Lymph Nodes: Clinicopathological Correlation and Prognostic Significance. *Gastrointestinal Disorders*. 2020;2(3):267-280. doi:10.3390/gidisord2030025

85. Angelucci A, Delle Monache S, Cortellini A, Di Padova M, Ficorella C. “Vessels in the Storm”: Searching for Prognostic and Predictive Angiogenic Factors in Colorectal Cancer. *Int J Mol Sci*. 2018;19(1):299. doi:10.3390/ijms19010299

86. Freire Valls A, Knipper K, Giannakouri E, et al. VEGFR1+ Metastasis–Associated Macrophages Contribute to Metastatic Angiogenesis and Influence Colorectal Cancer Patient Outcome. *Clinical Cancer Research*. 2019;25(18):5674-5685. doi:10.1158/1078-0432.CCR-18-2123

87. Ohnishi K, Komohara Y, Saito Y, et al. <scp>CD</scp> 169-positive macrophages in regional lymph nodes are associated with a favorable prognosis in patients with colorectal carcinoma. *Cancer Sci*. 2013;104(9):1237-1244. doi:10.1111/cas.12212

88. Saito Y, Fujiwara Y, Miyamoto Y, et al. <scp>CD169</scp> $^{+}$ sinus macrophages in regional lymph nodes do not predict mismatch-repair status of patients with colorectal cancer. *Cancer Med*. 2023;12(9):10199-10211. doi:10.1002/cam4.5747

89. Kim HJ, Park JH, Kim HC, Kim CW, Kang I, Lee HK. Blood monocyte-derived CD169+ macrophages contribute to antitumor immunity against glioblastoma. *Nat Commun*. 2022;13(1):6211. doi:10.1038/s41467-022-34001-5

90. Li C, Luo X, Lin Y, et al. A Higher Frequency of CD14+CD169+ Monocytes/Macrophages in Patients with Colorectal Cancer. *PLoS One*. 2015;10(10):e0141817. doi:10.1371/journal.pone.0141817

91. Kaprio T, Hagström J, Andersson LC, Haglund C. Tetraspanin CD63 independently predicts poor prognosis in colorectal cancer. *Histol Histopathol*. 2020;35(8):887-892. doi:10.14670/HH-18-209

92. Zadka Ł, Piotrowska A, Opalińska A, et al. Comparative analysis of exosome markers and extracellular vesicles between colorectal cancer and cancer-associated normal colonic mucosa. *Pol Arch Intern Med*. Published online June 26, 2020. doi:10.20452/pamw.15462

93. Titu S, Grapa CM, Mocan T, Balacescu O, Irimie A. Tetraspanins: Physiology, Colorectal Cancer Development, and Nanomediated Applications. *Cancers (Basel)*. 2021;13(22):5662. doi:10.3390/cancers13225662

Table S2

Key differences between Kupffer cell s in Normal liver and LM

**Feature, Function, Role**	**Normal Liver**	**Reference**	**Tumor microenvironment of metastasis**	**Reference**
Morphology	Kupffer cell (KCs) are amoeboid-shaped and are attached to the sinusoidal endothelial cells.<br>spindle or stellate-shaped cytoplasm, components of liver vascular walls<br>KCKC maintain a normal, non-activated state.	[1, 2]	KCs may become activated in response to tumor cells, leading to hypertrophy (enlargement) of the cells. Activated KCs may show increased cytoplasmic vacuoles<br>KCs and other macrophages were found to leave the sinusoids and migrate to sites of potential tumor development where they interacted with tumor cells and intimately wrapped their processes around fat storing cells	[2-4]
Phenotypic polarization	KCs typically exhibit an M1-like phenotype (CD80, CD86, Ly6C1) and Pan macrophages (CD68, CD14, CCR2, CD163)	[5-6]	KCs often undergo polarization towards an M2-like phenotype (CD36, LCD206, CD209, CD163)	[5-7]
Recruitment and polarization	KCs are maintained through self-renewal and local proliferation, with minimal recruitment from circulating monocytes	[5]	In colorectal cancer liver metastasis (CRC LM), there is increased recruitment of monocyte-derived macrophages, which differentiate into M2-like KCs.	[6, 8]
Phagocytic Activity	KCs exhibit robust phagocytic activity, clearing pathogens, cellular debris, and potentially tumor cells in the early stages of metastasis.	[9]	The phagocytic activity of KCs can be impaired or altered, potentially contributing to tumor cell survival and metastatic progression.	[4, 10]
Interactions with other cells	KCs interact with resident liver cells, such as hepatocytes and stellate cells	[9]	KCs interact with cancer cells, cancer-associated fibroblasts (CAFs), and other immune cells	[4, 10, 11]

Cytokine and Chemokine Production:	KCs produce a balanced array of cytokines and chemokines to maintain immune homeostasis and regulate inflammatory responses.	[4]	KCs often produce higher levels of immunosuppressive cytokines (e.g., IL-10, TGF-β) and pro-angiogenic factors (e.g., VEGF), promoting tumor growth and metastasis.	[4]
Functional roles	Crucial role in clearing pathogens, removing cellular debris, and maintaining liver homeostasis through their phagocytic and immunomodulatory functions	[4, 12]	Formation of premetastatic niches.<br>KCs can exhibit both pro-tumor and anti-tumor functions, depending on the stage of metastasis and the specific microenvironmental cues.<br>contribute to an immunosuppressive microenvironment	[4]

Abbreviations: CAFs: cancer-associated fibroblasts; CRC LM:colorectal cancer liver metastasis; KCs: Kupffer cellls;

References.

1. Dixon LJ, Barnes M, Tang H, Pritchard MT, Nagy LE. KCs in the Liver. In: *Comprehensive Physiology*. Wiley; 2013:785-797. doi:10.1002/cphy.c120026

2. Vekemans K, Braet F. Structural and functional aspects of the liver and liver sinusoidal cells in relation to colon carcinoma metastasis. *World J Gastroenterol*. 2005;11(33):5095. doi:10.3748/wjg.v11.i33.5095

3. Bonnardel J, T'Jonck W, Gaublomme D, et al. Stellate Cells, Hepatocytes, and Endothelial Cells Imprint the KC Identity on Monocytes Colonizing the Liver Macrophage Niche. *Immunity*. 2019;51(4):638-654.e9. doi:10.1016/j.immuni.2019.08.017

4. García-Pérez R, Ferrer Fábrega J, Varona-Bosque A, et al. Role of KCs in the progression of CRC liver metastases after the first stage of ALPPS. *Sci Rep*. 2018;8(1):8089. doi:10.1038/s41598-018-26082-4

5. Ali E, Červenková L, Pálek R, et al. Prognostic role of macrophages and mast cells in the microenvironment of hepatocellular carcinoma after resection. *BMC Cancer*. 2024;24(1):142. doi:10.1186/s12885-024-11904-8

6. Blériot C, Ginhoux F. Understanding the Heterogeneity of Resident Liver Macrophages. *Front Immunol*. 2019;10. doi:10.3389/fimmu.2019.02694

7. Jaitin DA, Adlung L, Thaiss CA, et al. Lipid-Associated Macrophages Control Metabolic Homeostasis in a Trem2-Dependent Manner. *Cell*. 2019;178(3):686-698.e14. doi:10.1016/j.cell.2019.05.054

8. Liu K. Pathomorphological study on location and distribution of KCs in hepatocellular carcinoma. *World J Gastroenterol*. 2003;9(9):1946. doi:10.3748/wjg.v9.i9.1946

9. Roy-Chowdhury N, Roy-Chowdhury J. Liver Physiology and Energy Metabolism. In: *Sleisenger and Fordtran's Gastrointestinal and Liver Disease*. Elsevier; 2010:1207-1225.e3. doi:10.1016/B978-1-4160-6189-2.00072-X

10. MATSUMURA H, KONDO T, OGAWA K, et al. KCs decrease metastasis of colon cancer cells to the liver in the early stage. *Int J Oncol*. 2014;45(6):2303-2310. doi:10.3892/ijo.2014.2662

11. Wen SW, Ager EI, Christophi C. Bimodal role of KCs during colorectal cancer liver metastasis. *Cancer Biol Ther*. 2013;14(7):606-613. doi:10.4161/cbt.24593

12. Thomson AW, Knolle PA. Antigen-presenting cell function in the tolerogenic liver environment. *Nat Rev Immunol*. 2010;10(11):753-766. doi:10.1038/nri2858
